# Benzoxazole Derivatives as Potent FXR and PPARα Dual Agonists With Anti‐Fibrotic and Metabolic Regulatory Effects

**DOI:** 10.1002/mco2.70442

**Published:** 2025-10-26

**Authors:** Mi‐Jeong Kim, Dong‐Gyun Han, Hyeon Seo Park, Sugyeong Ha, Sang Gyun Noh, Jeongwon Kim, Ji‐an Yoo, Byeong Moo Kim, Khas‐Erdene Battogtokh, Soohwan Oh, Youngmi Jung, Youngsuk Jung, Hae Young Chung, Hyung Ryong Moon, In‐Soo Yoon, Ki Wung Chung

**Affiliations:** ^1^ College of Pharmacy and Research Institute for Drug Development Pusan National University Busan Republic of Korea; ^2^ Department of Manufacturing Pharmacy and Research Institute for Drug Development College of Pharmacy Pusan National University Busan Republic of Korea; ^3^ College of Pharmacy and Interdisciplinary Major Program in Bio Medical and Data Science Convergence Korea University Sejong Republic of Korea; ^4^ Department of Biological Sciences College of Natural Science Pusan National University Pusan Republic of Korea

**Keywords:** dual agonist, farnesoid X receptor (FXR), kidney fibrosis, liver fibrosis, peroxisome proliferator‐activated receptor alpha (PPARα)

## Abstract

Fibrotic disease involves excessive fibrous connective tissue accumulation in organs, leading to dysfunction and irreversible damage. Metabolic alterations can sometimes contribute to fibrosis development. This study aimed to develop dual agonists for farnesoid X receptor (FXR) and peroxisome proliferator‐activated receptor alpha (PPARα), targeting anti‐fibrosis and metabolic regulation. Benzoxazole derivatives were found to potently activate both FXR and PPARα in hepatocytes. Among them, MHY5396 showed the most potent effects with low EC_50_ values. MHY5396 reduced lipid synthesis and enhanced beta‐oxidation in hepatocytes, decreasing lipid accumulation. It also suppressed TGFβ‐induced fibrosis in hepatic stellate cells. In a methionine/choline‐deficient diet mouse model, MHY5396 reduced lipid accumulation, liver damage, and fibrosis. In a thioacetamide‐induced liver fibrosis model, MHY5396 had an anti‐fibrotic effect comparable to obeticholic acid, a potent FXR agonist. MHY5396 also significantly reduced inflammation and fibrosis in renal cells and a folic acid‐induced renal fibrosis mouse model. Pharmacokinetic studies showed that orally administered MHY5396 was well absorbed (*F* = 98.6%) and primarily metabolized by hepatic CYP1A2 with negligible urinary excretion. Overall, MHY5396, with dual FXR and PPARα agonist activity, exhibited significant anti‐fibrotic and metabolic regulatory properties in liver and kidney fibrosis models, presenting a novel therapeutic potential for fibrotic diseases.

## Graphical abstract

MHY5396, a benzoxazole derivative, functions as a potent dual FXR and PPARα agonist. It exhibits significant anti‐fibrotic and metabolic regulatory effects, effectively improving liver and kidney fibrosis in experimental models. These findings highlight its potential as a therapeutic candidate for fibrosis and metabolic disorders.

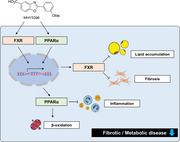

## 1 Introduction

Fibrosis is a pathological process characterized by abnormal accumulation of fibrous connective tissue, primarily consisting of collagen and elastic fibers, within an organ or tissue [1]. Fibrosis can be triggered by various factors, including tissue injury, trauma, infection, genetic factors, and environmental factors. Accumulation of fibrous tissue is an essential aspect of wound healing in response to damage. However, excessive accumulation can lead to tissue remodeling, resulting in scar tissue formation and disruption of the normal structure and function of damaged areas [2]. Inflammation is often the initial trigger of fibrosis, as it activates fibroblasts and other cell types involved in the production of extracellular matrix components. Chronic diseases with prolonged inflammatory responses can lead to unresolved tissue damage owing to the accumulation of scar tissue [3]. Fibrosis can affect multiple organs, such as the liver, kidneys, lungs, heart, and skin, and is closely linked to diseases such as cirrhosis, renal fibrosis, pulmonary fibrosis, and scleroderma [4]. Fibrosis imposes a significant disease burden, making the need for effective anti‐fibrotic therapies crucial. However, due to the complex and dynamic nature of fibrotic disorders, no therapies are currently available to prevent or reverse fibrosis. The field of anti‐fibrotic drug development and management faces several challenges, but also presents significant opportunities.

Considerable advancements have been achieved in unraveling the molecular mechanisms and pathobiology underlying fibrosis. Recent studies indicate that alterations in lipid metabolism, a common mechanism and core pathway in various fibrotic disorders, play a key role in the transition from normal wound healing to chronic fibrosis [5, 6]. The most well‐established link between lipid metabolism and fibrosis is the direct toxic effect of fatty acids on the liver [7]. Lipid accumulation and damaged hepatocytes directly increase fibrotic responses in stellate cells by releasing inflammatory cytokines and fibrotic mediators, further aggravating the liver pathology [8, 9]. Other lipid metabolic processes including fatty acid oxidation (FAO) and lipid synthesis mediated by acetyl‐CoA carboxylase are directly associated with fibrosis development [10, 11]. Additionally, growing evidence highlights the role of lipid metabolism in renal fibrosis development [12]. Defective FAO in kidney disease is directly associated with lipid accumulation and ATP depletion in the renal tubule epithelial cells, leading to fibrosis [13]. SREBP‐mediated lipid accumulation leads to increased lipotoxicity and fibrosis in the kidneys [14]. The importance of lipid metabolism in the development of fibrosis has also been implicated in myocardial and pulmonary fibrosis [5]. Thus, targeting lipid metabolism offers new insights into the pathogenesis of fibrotic diseases, paving the way for developing drugs to prevent and treat fibrosis.

The farnesoid X receptor (FXR) functions as a ligand‐activated nuclear receptor, specifically for bile acids, and acts as a transcription factor [15]. FXR plays a major role in maintaining biliary homeostasis and cholesterol metabolism and in supervising various other metabolic functions, including lipid metabolism [16]. Activation of FXR by bile acids lowers triglyceride (TG) levels via a pathway involving SHP and SREBP1c in the liver [17]. A recent report showed that FXR activation protects against liver lipid accumulation by reducing *Scd1, Dgat2*, and *Lpin1* expression in the liver [18]. In addition, FXR activation has been implicated in the development of liver fibrosis. The nuclear receptor, SHP, mediates the inhibition of hepatic stellate cells (HSCs) by FXR, thereby protecting against liver fibrosis [19]. Treatment with various FXR agonists has shown protective effects in several animal models of liver fibrosis [20, 21]. Additionally, FXR activation has been implicated in kidney lipid metabolism and fibrosis [22]. FXR activation attenuates kidney injury by increasing FAO, suppressing lipid synthesis, and reducing fibrotic and inflammatory responses in the kidneys [23, 24].

Peroxisome proliferator‐activated receptor alpha (PPARα) is a nuclear receptor protein that functions as a ligand‐activated transcription factor by binding to peroxisome proliferator response elements (PPREs). PPARα plays an important role in regulating genes involved in lipid metabolism, especially in FAO [25]. In addition to FAO, its effects on other metabolic processes such as inflammation and fibrosis have been extensively studied [26]. In various liver disease models, the administration of a PPARα agonist has been shown to not only reduce lipid accumulation but also alleviate fibrosis. Pawlak et al. proposed that the transrepressive activity of PPARα is both necessary and sufficient to prevent liver fibrosis in mice [10]. However, PPARα agonists have failed in clinical trials in patients with liver disease, highlighting the challenge of translating findings from animal models to human diseases. Further studies are focusing on the development of compounds with selective activity for PPARα or synergistic agonism targeting both PPARα and other pathways [27]. The role of PPARα in kidney fibrosis has also been demonstrated in independent animal models. The importance of FAO governed by PPARα is directly associated with renal fibrosis development [13]. Altered PPARα and FAO are also associated with age‐related kidney fibrosis, and restoring FAO by activating PPARα reduces lipid accumulation and prevents kidney fibrosis [28].

Based on the previously reported role of FXR and PPARα and the potency of their agonists in different models of fibrosis, we aimed to develop and evaluate novel FXR and PPARα dual agonists. The effect of the selected compound was evaluated in two different organs, the kidney and liver, where the prevalence of fibrosis is the highest. The regulatory and anti‐fibrotic properties of the selected compounds were evaluated in hepatocytes and stellate cells. The in vivo effects of the selected compounds were evaluated in two liver disease models. The efficacy of the selected compounds was further evaluated in kidney cells and a kidney fibrosis model. Finally, based on their efficacy in animal models, drug metabolism and pharmacokinetic properties of the selected compounds were evaluated.

## 2 Results

### 2.1 Evaluation of Benzoxazole Derivatives as Potent FXR and PPARα Dual Agonists

**FIGURE 1:**
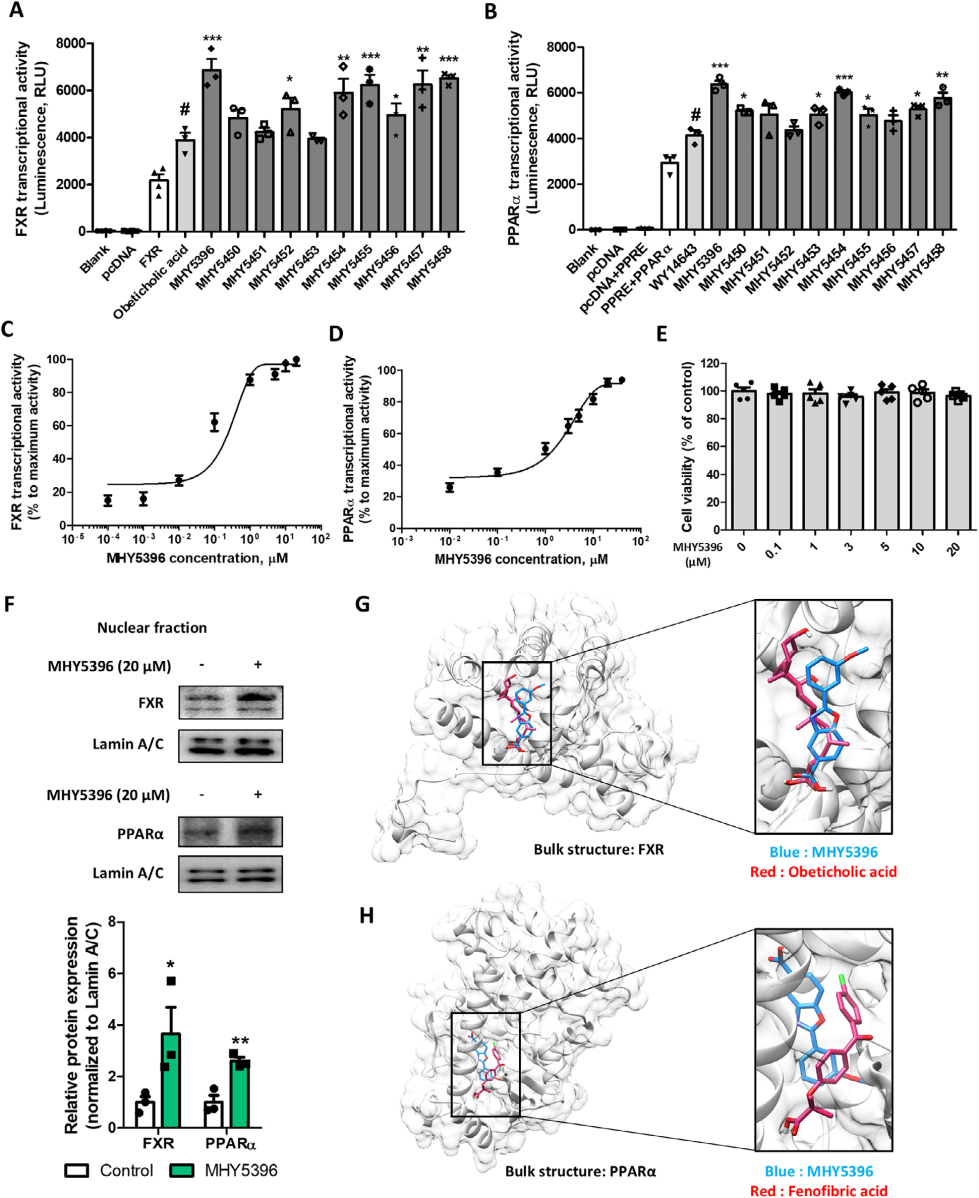
Evaluation of benzoxazole derivatives as potent farnesoid X receptor (FXR) and peroxisome proliferator‐activated receptor alpha (PPARα) dual agonists in HepG2 hepatocytes. (A) Effect of benzoxazole compounds on FXR transcriptional activity was assessed using a luciferase system (*n* = 3). #*p* < 0.05 compared to the FXR group. *p < 0.05, **p < 0.005, ***p < 0.001 compared to the obeticholic acid (OCA)‐treated group. (B) Effect of benzoxazole compounds on PPARα transcriptional activity was assessed using PPRE luciferase system (*n* = 3). #*p* < 0.05 compared to the PPRE+PPARα group. *p < 0.05, **p < 0.005, ***p < 0.001 compared to the WY14643‐treated group. (C) Dose‐dependent effects of MHY5396 on FXR transcriptional activity were determined using the luciferase system. (D) Dose‐dependent effects of MHY5396 on PPARα transcriptional activity were determined using PPRE luciferase system. (E) Evaluation of MHY5396 cytotoxicity based on concentration (*n* = 5). (F) FXR and PPARα expression after MHY5396 treatment in the nuclear fraction of cells. Lamin A/C was used as the internal control for the nuclear fraction. Relative protein expressions were quantified using densitometry (*n* = 3). *p < 0.05, **p < 0.005 compared to the control group. (G) 3D docking image of MHY5396 (blue) and OCA with FXR. (H) 3D docking image of MHY5396 (blue) and fenofibric acid with PPARα.

Initially, we screened a potential agonist of FXR and PPARα, which was synthesized based on an FXR agonist. Through this screening, we found that benzoxazole derivatives commonly exhibited a dual agonist effect (Figure S1). The transcriptional activities of FXR and PPARα in HepG2 hepatocytes were compared with those of their respective agonists, OCA and WY14643, respectively (Figure 1A,B). Most MHYs exhibited higher transcriptional activity than agonist compounds, implying that benzoxazole derivatives act as dual agonists for FXR and PPARα. Among the screened compounds, MHY5396 demonstrated the highest FXR and PPARα transcriptional activity and was selected for further evaluation. MHY5396 enhanced the transcriptional activity of both FXR (EC_50_ = 0.074 µM) and PPARα (EC_50_ = 3.77 µM) in a dose‐dependent manner (Figure 1C,D) and did not show significant cytotoxicity up to 20 µM in HepG2 hepatocytes (Figure 1E). We next examined the nuclear protein expression of FXR and PPARα in cells and confirmed that MHY5396 significantly increased their expression in the nucleus (Figure 1F). Thermal shift analysis was further implemented to confirm the direct interaction between protein and ligand. The results showed that the melting temperatures of FXR and PPARα LBDs were altered upon treatment with OCA, fenofibric acid, or MHY5396. These results suggest that the ligands bind to the LBDs and affect their thermal stability (Figure S2A,B). Docking simulations were performed to assess binding affinities between proteins and ligands. The predicted binding modes are shown in Figure 1G,H. According to AutoDock Vina, MHY5396 showed stronger binding to FXR than the positive control (OCA), while it also showed higher affinities for PPARα than fenofibric acid in both AutoDock Vina and LeDock (Table S1). Based on these results, pharmacophore analysis predicted key interactions, visualized in Figure S3 and detailed in Table S2. DiffDock‐L simulations further supported polar contacts between FXR (THR296, TRP458, ARG459) and MHY5396, and between PPARα (SER280, THR314) and MHY5396 (Figure S4). Collectively, these data suggested that benzoxazole derivatives were potent FXR and PPARα dual agonists and that MHY5396 was an effective dual agonist without cytotoxicity.

### 2.2 MHY5396 Modulates Lipid Metabolism in Hepatocytes and Fibrotic Response in Liver Stellate Cells

**FIGURE 2:**
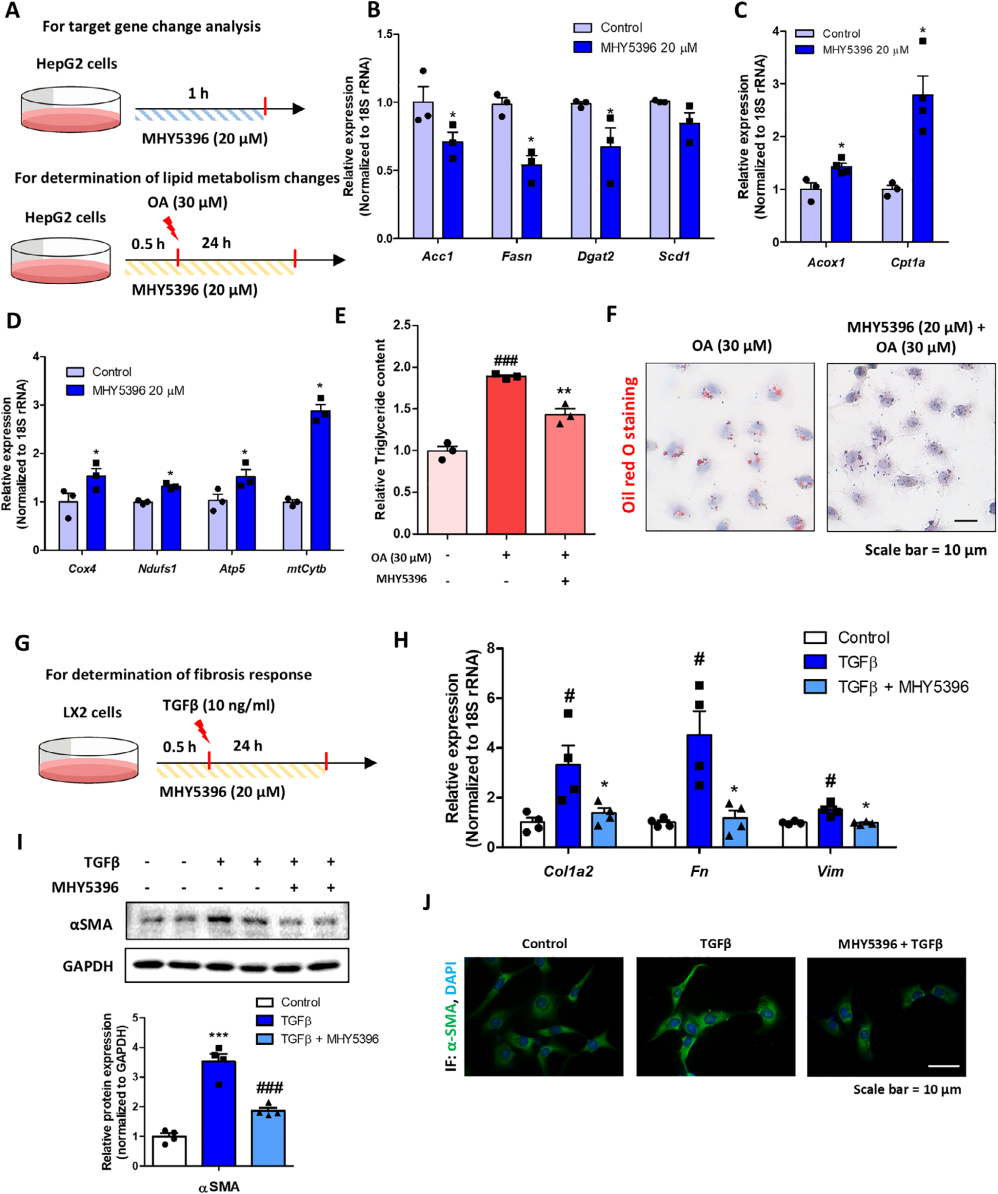
MHY5396 regulates lipid metabolism in HepG2 hepatocytes and fibrosis response in LX2 stellate cells. (A) Schematic diagram of the experiment to confirm genetic changes caused by MHY5396 treatment and alterations in oleic acid (OA)‐induced lipid metabolism by MHY5396 treatment. (B) Relative mRNA levels of lipid synthesis genes (*Acc1, Fasn, Dgat2*, and *Scd1*) in MHY5396‐treated HepG2 cells (*n* = 3). The results are quantified as ratios of 18 S rRNA. *p < 0.05 compared to the control group. (C) Relative mRNA levels of β‐oxidation‐related genes (*Acox1* and *Cpt1a*) in MHY5396‐treated HepG2 cells (*n* = 3). The results are quantified as ratios of 18S rRNA. *p < 0.05 compared to the control group. (D) Relative mRNA levels of mitochondria‐related genes (*Cox4, Ndufs1, Atp5*, and *mtCytb*) in MHY5396‐treated HepG2 cells (*n* = 3). The results are quantified as ratios of 18S rRNA. *p < 0.05 compared to the control group. (E) Relative triglyceride (TG) levels in OA with or without MHY5396 treatment of HepG2 cells (*n* = 3). ###*p* < 0.001 compared to the control group. **p < 0.005 compared to the OA‐treated group. (F) Representative images of Oil red O (ORO) staining of HepG2 cells. (G) Experimental scheme to evaluate the effect of MHY5396 on the TGFβ‐induced fibrotic response in LX2 cells. (H) Relative mRNA levels of fibrosis‐related genes (*Col1a2, Fn*, and *Vim*) in TGFβ‐treated LX2 cells with or without MHY5396 treatment (*n* = 4). The results are quantified as ratios of 18S rRNA. #*p* < 0.05 compared to the control group. *p < 0.05 compared to the TGFβ‐treated group. (I) Protein levels of αSMA determined in TGFβ‐treated LX2 cells with or without MHY5396 treatment. GAPDH was used as the loading control (*n* = 4). Relative protein expressions were quantified using densitometry. ***p < 0.001 compared to the control group; ###*p* < 0.001 compared to the TGFβ‐treated group. (J) Representative immunofluorescence (IF) images of αSMA expression (green) in TGFβ‐treated LX2 cells with or without MHY5396 treatment. The nuclei were counterstained with DAPI.

Based on the role of FXR and PPARα in liver biology, we evaluated the effect of MHY5396 under in vitro conditions using cell experiments. Using HepG2 hepatocytes, we evaluated lipid metabolism regulatory effects of MHY5396 (Figure 2A). MHY5396 treatment significantly decreased lipid synthesis‐associated gene expression (*Acc1, Fasn, Dgat2*, and *Scd1*) and increased lipid β‐oxidation‐associated gene expression (*Acox1* and *Cpt1a*) in HepG2 hepatocytes (Figure 2B,C). MHY5396 also induced mitochondrial oxidative phosphorylation (OXPHOS) gene expression (*Cox4, Ndufs1, Atp5*, and *mtCytb*) in cells (Figure 2D). We further evaluated its role in oleic acid (OA)‐induced lipid accumulation in cells. MHY5396 pretreatment significantly blocked OA‐induced lipid accumulation in cells, as determined by TG quantification and ORO staining (Figure 2E,F). The effect of MHY5396 was confirmed in another liver cell line, AC2F. Similar to the results observed in HepG2 cells, MHY5396 showed regulatory effects on lipid metabolism in AC2F cells (Figure S5A–E). Next, we evaluated the anti‐fibrotic effect of MHY5396 on LX2 liver stellate cells (Figure 2G). MHY5396 treatment significantly blocked TGFβ‐induced fibrosis‐related gene expression (*Col1a2, Fn*, and *Vim*) (Figure 2H). MHY5396 also reduced TGFβ‐induced αSMA protein levels in stellate cells (Figure 2I,J). By conducting experiments with single agonists, we determined that lipid regulatory effects were primarily mediated by PPARα agonism in hepatocytes, whereas the anti‐fibrotic effects were reliant on FXR activation in stellate cells (Figure S6). Collectively, these findings suggested that MHY5396 effectively inhibited hepatic lipid accumulation and fibrosis under in vitro conditions.

### 2.3 MHY5396 Alleviates Methionine Choline‐Deficient Diet (MCD) and High‐Fat Diet (HFD)‐Induced Lipid Accumulation and Fibrosis in Liver

**FIGURE 3:**
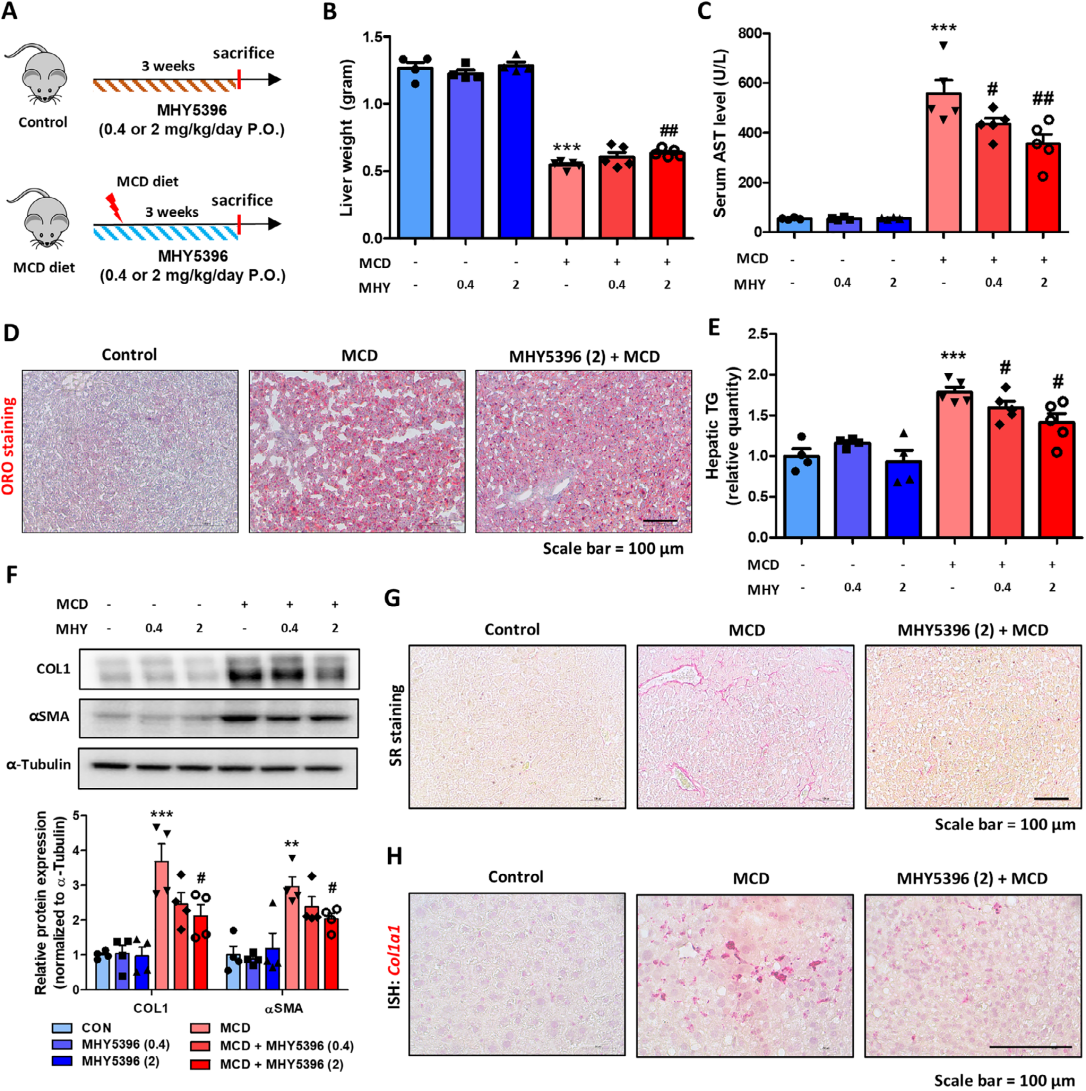
MHY5396 alleviates methionine/choline‐deficient diet‐induced lipid accumulation and fibrosis in the liver. (A) Experimental scheme to assess the effect of MHY5396 in a methionine/choline‐deficient (MCD) diet‐induced hepatic lipid accumulation and fibrosis model (*n* = 6–8). (B) Final mouse liver weight on the last day of the experiment. ***p < 0.001 compared to the control mouse group. ##*p* < 0.005 compared to the MCD diet mouse group. (C) Serum AST levels in MCD diet mice with or without MHY5396 treatment. ***p < 0.001 compared to the control mouse group. #*p* < 0.05, ##*p* < 0.005 compared to the MCD diet mouse group. (D) Representative ORO staining of mouse liver tissue sections. (E) Hepatic TG levels in the liver of MCD diet mice with or without MHY5396 treatment. ***p < 0.001 compared to the control mouse group. #*p* < 0.05, ##*p* < 0.005 compared to the MCD diet mouse group. (F) COL1 and αSMA protein levels were determined in the liver of MCD diet mice with or without MHY5396 treatment. α‐Tubulin was used as the loading control. Relative protein expressions were quantified using densitometry. **p < 0.005, ***p < 0.001 compared to the control group. #*p* < 0.05 compared to the MCD group. (G) Representative Sirius red (SR) staining of mouse liver tissue sections. (H) Representative in situ hybridization (ISH) images detected with *Col1a1* (red) probe in the liver of MCD diet mice with or without MHY5396 treatment.

To evaluate the in vivo effects of MHY5396, we first confirmed that oral administration of MHY5396 alters the expression of genes involved in lipid metabolism in the livers of treated mice, consistent with our in vitro findings (Figure S7). Next, we employed an MCD diet‐induced liver injury model to assess the effect of MHY5396 on hepatic lipid accumulation (Figure 3A). The MCD diet significantly reduced liver and body weights in mice (Figure 3B, Figure S8A). The high‐dose MHY5396 group (2 mg/kg) showed a small decrease in liver weight than the control group (Figure 3B). Serum AST and ALT levels were significantly increased by the MCD diet and MHY5396 effectively blocked this increase (Figure 3C, Figure S8B). Hematoxylin and eosin (H&E) staining revealed histological changes among the groups. The MCD diet significantly increased lipid droplet positioning and damaged hepatocytes in the liver, whereas the MHY5396 group showed fewer changes in the liver (Figure S8C). ORO staining and TG quantification revealed lower lipid accumulation in the MHY5396 group than that in the MCD group (Figure 3D,E). The MHY5396 group showed lower expression of genes associated with lipid synthesis (*Srebp1, Srebp2, Pparr, Acca, Fasn*, and *Scd1*) than the MCD group (Figure S8D). We further compared fibrotic responses in the liver. The MHY5396 group showed less fibrosis‐related gene expression (*Tgfb1, Acta2, Fn*, and *Col1a1*) and protein expression (COL1 and αSMA) than the MCD group (Figure 3F, Figure S8E). Histological analysis performed using Sirius Red (SR) staining and *Col1a1* and *Vim* ISH also showed fewer fibrotic regions in the MHY5396 group than in the MCD group (Figure 3G,H, Figure S8F). We also evaluated the toxicity of MHY5396. Administration of MHY5396 for 3 weeks did not cause any liver toxicity, as evidenced by serum AST and ALT levels (Figure 3C, Figure S8B). Additionally, MHY5396 did not exhibit a cell proliferative phenotype, a commonly detected adverse effect of PPARα agonists. This was assessed by measuring the liver weight and expression of cell proliferation genes (Figure 3B, Figure S9A–C). The effect of MHY5396 on lipid metabolism was further investigated using an HFD‐induced steatosis model. MHY5396 effectively reduced lipid accumulation in the livers of HFD‐fed mice (Figure S10). These results suggested that MHY5396 effectively blocked MCD‐induced lipid accumulation and fibrosis in the liver.

### 2.4 MHY5396 Alleviates TAA‐Induced Liver Fibrosis

**FIGURE 4:**
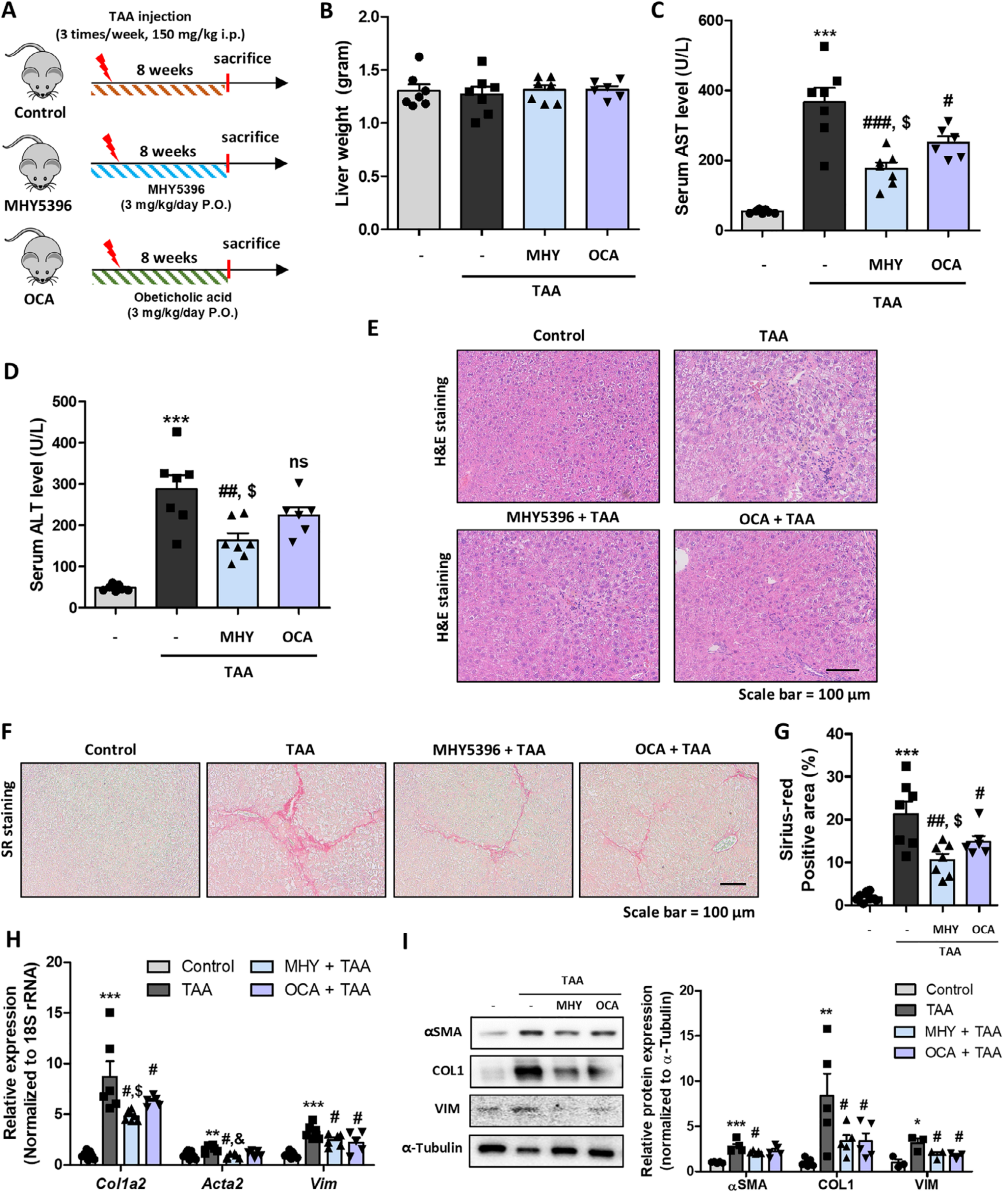
Comparison of anti‐fibrotic efficacy between MHY5396 and OCA in a thioacetamide‐induced liver fibrosis model. (A) Experimental scheme used to evaluate the effect of MHY5396 in a thioacetamide (TAA)‐induced hepatic fibrosis model (*n* = 6–7). (B) Final mouse liver weight on the last day of the experiment. (C) Serum AST levels in TAA‐treated mice with or without MHY5396 treatment. ***p < 0.001 compared to the control mouse group. #*p* < 0.05, ###*p* < 0.001 compared to the TAA‐treated group. $*p* < 0.05 compared to the OCA‐treated group. (D) Serum ALT levels in TAA‐treated mice with or without MHY5396 treatment. ***p < 0.001 compared to the control mouse group. ##*p* < 0.005 compared to the TAA‐treated group. $*p* < 0.05 compared to the OCA‐treated group. (E) Representative H&E staining of mouse liver tissue sections. (F) Representative SR staining of mouse liver tissue sections. (G) Positive areas for SR staining were calculated to determine the extent of hepatic fibrosis in each experimental group. ***p < 0.001 compared to the control mouse group. #*p* < 0.05, ##*p* < 0.0‐5 compared to the TAA‐injected mouse group. $*p* < 0.05 compared to the OCA‐treated group. (H) Relative mRNA levels of hepatic fibrosis‐related genes (*Col1a2, Acta2*, and *Vim*) with or without MHY5396 treatment. The results are quantified as ratios of 18S rRNA. **p < 0.005, ***p < 0.001 compared to the control mouse group. #*p* < 0.05 compared to the TAA‐injected mouse group. $*p* < 0.05 compared to the OCA‐treated group. (I) αSMA, Col1a1, and vimentin protein levels were determined in TAA‐injected mouse livers with or without MHY5396 treatment. α‐Tubulin was used as the loading control. Relative protein expressions were quantified using densitometry. *p < 0.05, **p < 0.005, ***p < 0.001 compared to the control group. #*p* < 0.05 compared to the TAA‐treated group.

We further tested the effects of MHY5396 in a TAA‐induced liver fibrosis model. In this experiment, we compared the effect of MHY5396 with that of the well‐known FXR activator, OCA. TAA treatment for 8 weeks did not result in significant changes in the liver weight (Figure 4A,B). Serum AST and ALT levels were significantly increased in the TAA group, whereas the MHY5396 group showed a significantly lower increase in AST and ALT levels (Figure 4C,D). Although the OCA group also showed a smaller increase in AST levels than that in the TAA group, the increase was still higher than that in the MHY5396 group (Figure 4C). H&E staining showed damaged hepatocytes in the TAA group, whereas the MHY5396 and OCA groups showed fewer damaged hepatocytes in the liver (Figure 4E). TAA increased the number of fibrotic regions, as detected by SR staining, and the MHY5396 group showed less fibrosis in the liver (Figure 4F,G). The expression of genes related to fibrosis (*Col1a2, Acta2*, and *Vim*) significantly increased in the TAA group, whereas MHY5396 treatment effectively blocked this increase (Figure 4H). The protein expression of COL1, αSMA, and VIM also showed a tendency similar to that of their gene expression (Figure 4I). We further tested treatment protocols in an animal model of liver fibrosis (Figure S11A). Post‐treatment with MHY5396, administered two weeks after TAA injection, significantly reduced liver damage and fibrosis compared to the untreated control group (Figure S11A–F). Although MHY5396 and OCA demonstrated protective effects against fibrosis in the TAA model, MHY5396 provided greater protection.

### 2.5 MHY5396 Exerts Anti‐Inflammatory Effects in Renal Epithelial Cells and Mitigates Fibrotic Response in Renal Fibroblasts

**FIGURE 5:**
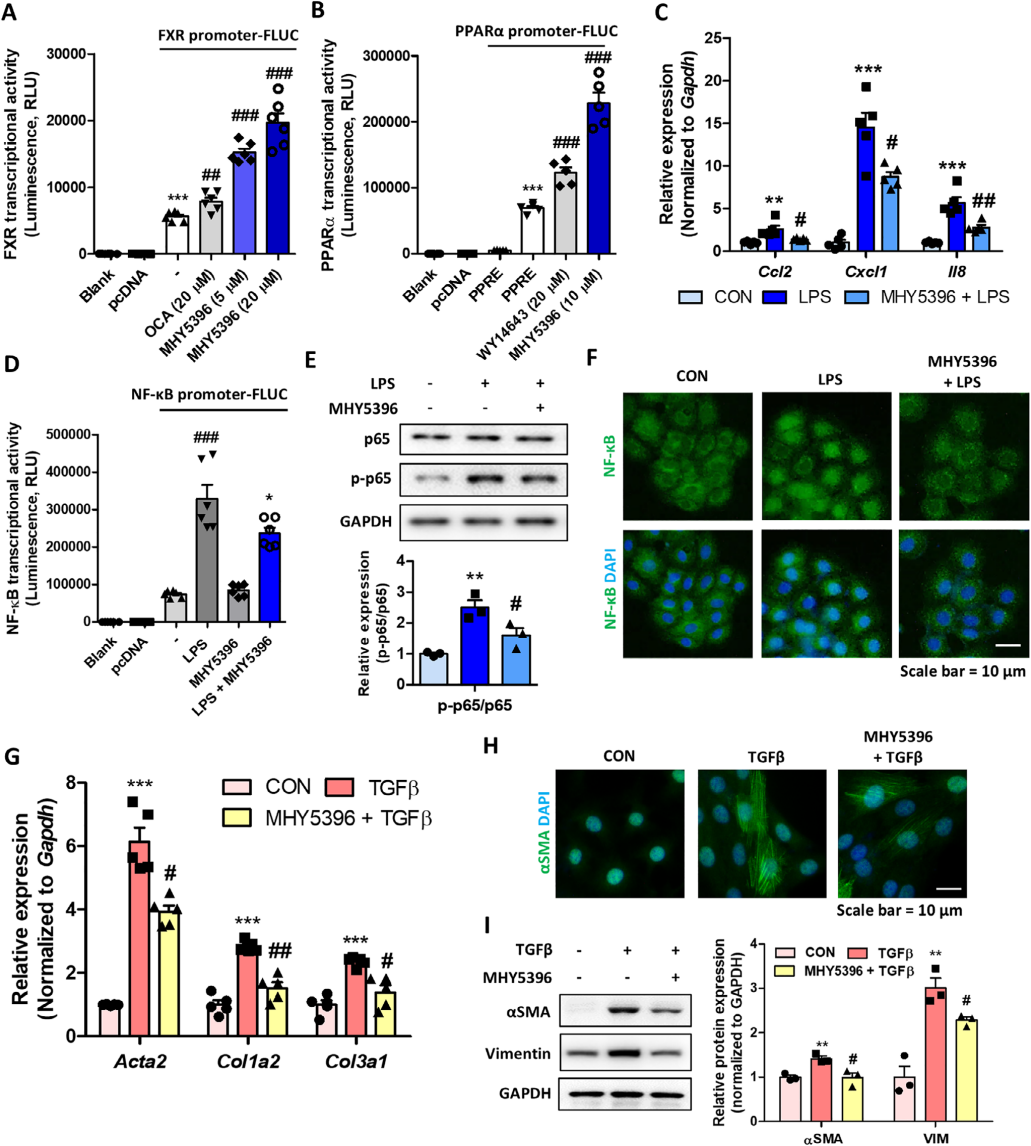
MHY5396 exerts anti‐inflammatory effects in renal epithelial cells and mitigates fibrotic responses in renal fibroblasts. (A) Effect of MHY5396 on FXR transcriptional activity was measured using the luciferase system in NRK52E cells (*n* = 6). ***p < 0.001 compared to the pcDNA group. ##*p* < 0.005, ###*p* < 0.001 compared to the FXR promoter only group. (B) Effect of MHY5396 on PPARα transcriptional activity was measured using the PPRE luciferase system in NRK52E cells (*n* = 5). ***p < 0.001 compared to the PPRE group. ###*p* < 0.001 compared to the PPRE+PPARα group. (C) Relative mRNA levels of chemokine genes (*Ccl2, Cxcl1*, and *Il8*) in lipopolysaccharide (LPS)‐treated NRK52E cells with or without MHY5396 (20 µM) treatment (*n* = 5). **p < 0.005, ***p < 0.001 compared to the control group. #*p* < 0.05, ##*p* < 0.005 compared to the LPS‐treated group. (D) NF‐κB transcriptional activity was assessed using the luciferase system in LPS‐treated NRK52E cells with or without MHY5396 (20 µM) treatment (*n* = 6). ###*p* < 0.001 compared to the NF‐κB group. *p < 0.05 compared to the LPS‐treated group. (E) Protein levels of p65 and p‐p65 were determined in LPS‐treated NRK52E cells with or without MHY5396 (20 µM) treatment (*n* = 3). GAPDH was used as the loading control. **p < 0.001 compared to the control group. # *p* <0.05 compared to the LPS‐treated group. (F) Representative IF images of p65 expression (green) in LPS‐treated NRK52E cells with or without MHY5396 (20 µM) treatment. The nuclei were counterstained with DAPI. (G) Relative mRNA levels of fibrosis‐related genes (*Acta2, Col1a2*, and *Col3a1*) in TGFβ‐treated NRK49F cells with or without MHY5396 (20 µM) treatment (*n* = 5). ***p < 0.001 compared to the control group. #*p* < 0.05, ##*p* < 0.005 compared to the TGFβ‐treated group. (H) Representative IF images of αSMA expression (green) in TGFβ‐treated NRK49F cells with or without MHY5396 (20 µM) treatment. (I) Protein levels of αSMA and vimentin in TGFβ‐treated NRK49F cells with or without MHY5396 (20 µM) treatment (*n* = 3). GAPDH was used as the loading control. Relative protein expressions were quantified using densitometry. *p < 0.05, **p < 0.005, ***p < 0.001 compared to the control group. #*p* < 0.05 compared to the TGFβ‐treated group.

Activation of FXR and PPARα has been reported to have beneficial effects in kidney fibrosis models. Based on previous reports, we tested whether MHY5396 exerted anti‐fibrotic effects in a kidney model of fibrosis. We used renal tubule epithelial cells (NRK52E) to examine the anti‐inflammatory properties of MHY5396. In NRK52E cells, MHY5396 effectively increased FXR and PPARα transcriptional activity, similar to that observed in liver cells (Figure 5A,B), whereas no cytotoxicity was observed in these cells (Figure S12A). MHY5396 significantly reduced LPS‐induced chemokine (*Ccl2, Cxcl1*, and *Il8*) expression in cells (Figure 5C). We further tested whether MHY5396 directly reduces NF‐κB signaling in the cells. MHY5396 significantly reduced transcriptional activity of NF‐κB and phosphorylation of p65 in the cells (Figure 5D,E). Immunofluorescence staining further showed low LPS‐induced translocation of p65 into the nucleus (Figure 5F). These results suggest that MHY5396 shows anti‐inflammatory effects in renal epithelial cells by inhibiting the NF‐κB signaling pathway.

The anti‐fibrotic potential of MHY5396 was further assessed using NRK49F fibroblasts. Stimulation of NRK49F cells with TGFβ1 induced the expression of fibrosis‐related genes (*Acta2, Col1a2*, and *Col3a1*), and their expression was blocked by MHY5396 pretreatment (Figure 5G). The protein expression of αSMA and vimentin was analyzed. TGFβ significantly induced protein expression of αSMA and vimentin in fibroblasts, and MHY5396 pretreatment significantly reduced their expression in the cells (Figure 5H,I). FXR activation has been shown to reduce the fibrotic response through Smad transcription factor modulation, and we further examined Smad expression and activation. However, MHY5396 treatment did not affect Smad2/3 expression or phosphorylation in the fibroblasts (Figure S12B). These data suggest that MHY5396 mitigates TGFβ‐induced fibrosis response in renal fibroblasts.

### 2.6 MHY5396 Mitigates Renal Damage and Fibrosis Induced by Folic Acid (FA) in Mice

**FIGURE 6:**
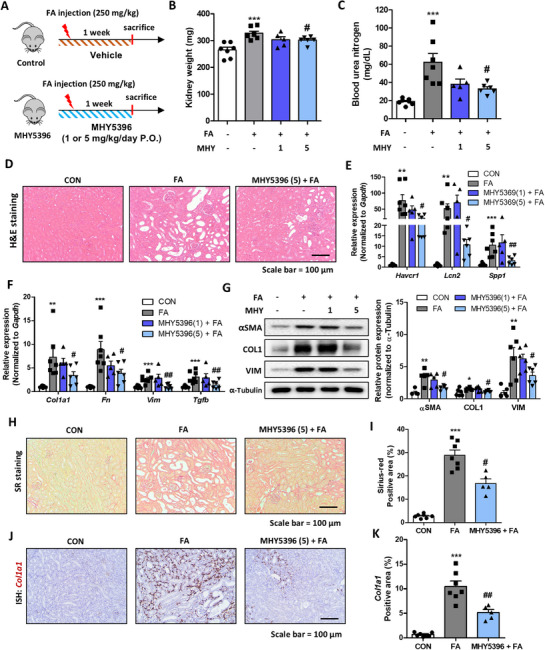
MHY5396 mitigates folic acid‐induced renal fibrosis in mice. (A) Experimental scheme to assess the effect of MHY5396 in folic acid (FA)‐induced renal fibrosis model (*n* = 5–7). (B) Final mouse kidney weight on the last day of the experiment. ***p < 0.001 compared to the control mouse group. #*p* < 0.05 compared to the FA‐injected mouse group. (C) Blood urea nitrogen (BUN) levels in FA‐injected serum with or without MHY5396 treatment. ***p < 0.001 compared to the control mouse group. #*p* < 0.05 compared to the FA‐injected mouse group. (D) Representative images of H&E staining of mouse kidney tissue sections. (E) Relative mRNA levels of kidney damage‐related genes (*Havcr1, Lcn2*, and *Spp1*) in FA‐injected mouse kidneys with or without MHY5396 treatment. **p < 0.005, ***p < 0.001 compared to the control mouse group. #*p* < 0.05, ##*p* < 0.005 compared to the FA‐injected mouse group. (F) Relative mRNA levels of kidney fibrosis‐related genes (*Col1a1, Fn, Vim*, and *Tgfb*) in FA‐injected mouse kidneys with or without MHY5396 treatment. **p < 0.005, ***p < 0.001 compared to the control mouse group. #*p* < 0.05, ##*p* < 0.005 compared to the FA‐injected mouse group. (G) Protein levels of αSMA, COL1, and VIM in FA‐injected mouse kidneys with or without MHY5396 treatment. α‐Tubulin was used as the loading control. Relative protein expressions were quantified using densitometry. *p < 0.05, **p < 0.005 compared to the control group. #*p* < 0.05 compared to the FA‐treated group. (H) Representative images of SR staining of mouse kidney tissue sections. (I) Positive areas from SR staining were quantified to determine the degree of kidney fibrosis in each experimental group. ***p < 0.001 compared to the control mouse group. #*p* < 0.05 compared to the FA‐injected mouse group. (J) Representative ISH images detected with *Col1a1* (brown) probe in FA‐injected mouse kidneys with or without MHY5396 treatment. (K) Positive areas for ISH were calculated to determine the extent of fibrosis in each experimental group. ***p < 0.001 compared to the control mouse group. ##*p* < 0.005 compared to the FA‐injected mouse group.

Based on the in vitro experimental results, we applied MHY5396 to an FA‐induced kidney fibrosis mouse model (Figure 6A). FA treatment slightly increased kidney weight, whereas a high dose of MHY5396 (5 mg/kg) significantly reduced kidney weight compared with FA‐treated kidneys (Figure 6B). In the FA‐injected group, BUN levels were significantly increased, but high doses of MHY5396 significantly reduced this FA‐induced increase (Figure 6C). H&E staining revealed increased tubule dilation in the FA group, whereas MHY5396 treatment reduced tubule dilation in the kidneys (Figure 6D). We evaluated the expression of kidney damage‐related genes (*Havcr1*, *Lcn2*, and *Spp1*) and found that MHY5396 treatment significantly reduced their expression in the kidneys (Figure 6E). The extent of fibrosis was assessed by various biochemical methods. The expression of fibrosis‐related genes (*Col1a1*, *Fn*, *Vim*, and *Tgfb*) was significantly reduced by MHY5396 treatment (Figure 6F). The protein levels of αSMA, COL1, and vimentin showed tendencies similar to their gene expression (Figure 6G). Histological analysis further confirmed reduced fibrosis in MHY5396‐treated kidneys. SR staining results indicated that FA treatment significantly increased collagen deposition in the kidney, whereas MHY5396 effectively reduced fibrosis development in the kidney (Figure 6H,I). ISH analysis further showed *Col1a1* expression in the interstitial region of fibrotic kidneys. MHY5396 reduced *Col1a1* expression in the kidneys (Figure 6J,K). Collectively, these results suggest that MHY5396 effectively mitigated FA‐induced renal damage and fibrosis in mouse kidneys.

### 2.7 MHY5396 Attenuates FA‐Induced Kidney Fibrosis by Suppressing Inflammation and Regulating Lipid Metabolism

**FIGURE 7:**
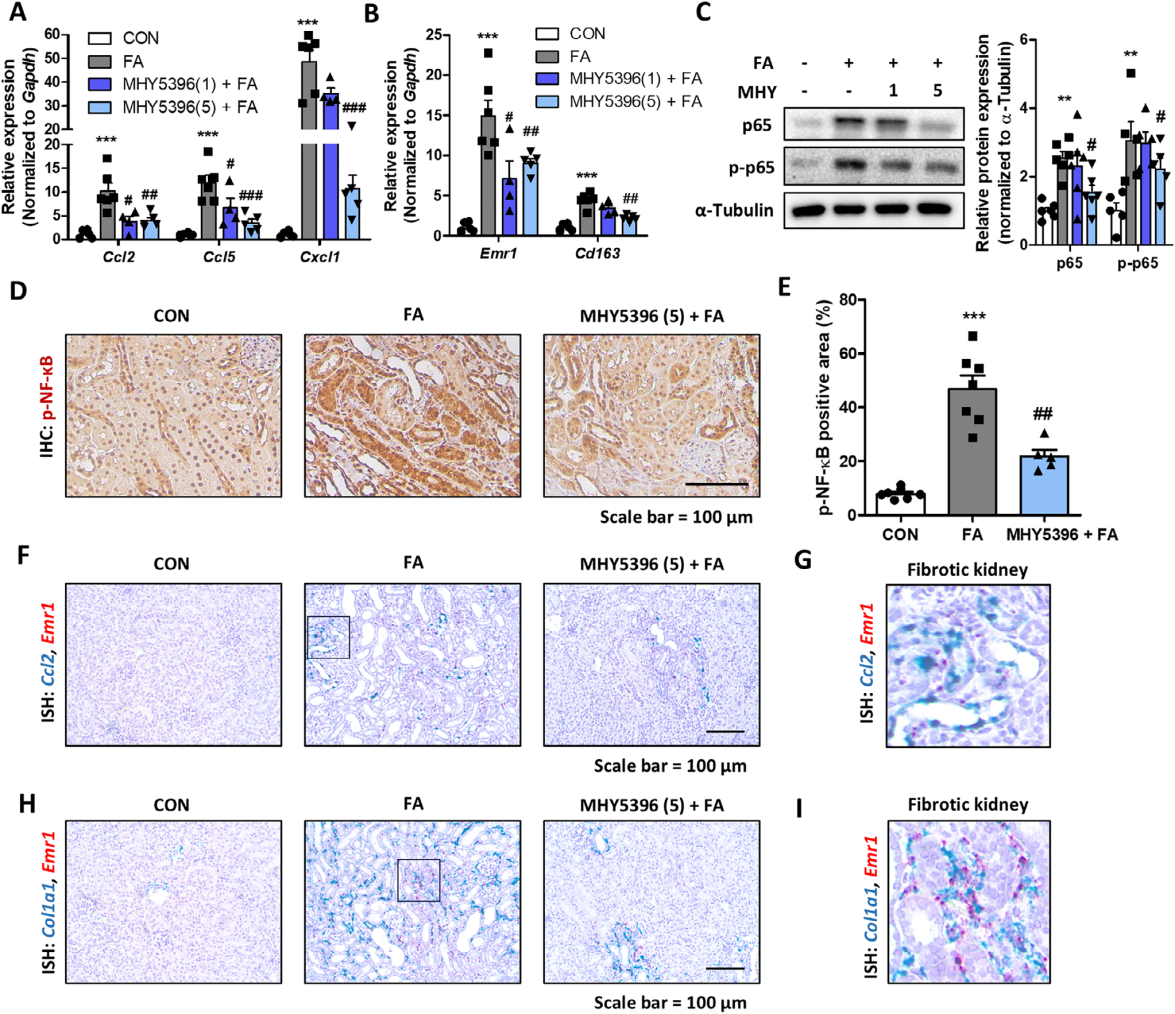
MHY5396 alleviates folic acid‐induced renal inflammation in mice. (A) Relative mRNA levels of kidney inflammation‐related genes (*Ccl2, Ccl5, and Cxcl1*) in FA‐injected mouse kidneys with or without MHY5396 treatment. ***p < 0.001 compared to the control mouse group. #*p* < 0.05, ##*p* < 0.005, ###*p* < 0.001 compared to the FA‐injected mouse group. (B) Relative mRNA levels of kidney macrophage‐related genes (*Emr1* and *Cd163*) in FA‐injected mouse kidneys with or without MHY5396 treatment. ***p < 0.001 compared to the control mouse group. #*p* < 0.05, ##*p* < 0.005 compared to the FA‐injected mouse group. (C) Protein levels of p65 and p‐p65 in FA‐injected mouse kidneys with or without MHY5396 treatment. α‐Tubulin was used as the loading control. Relative protein expressions were quantified using densitometry. **p < 0.005 compared to the control group. #*p* < 0.05 compared to the FA‐treated group. (D) Representative immunohistochemical (IHC) images of p‐p65 expression in FA‐injected mouse kidneys with or without MHY5396 treatment. (E) Positive areas in IHC were quantified to determine the extent of inflammation in each experimental group. ***p < 0.001 compared to the control mouse group. ##*p* < 0.005 compared to the FA‐injected mouse group. (F and G) Representative ISH images detected with *Ccl2* (blue) and *Emr1* (red) probes in FA‐injected mouse kidneys with or without MHY5396 treatment. (H and I) Representative ISH images detected with *Col1a1* (blue) and *Emr1* (red) probes in FA‐injected mouse kidneys with or without MHY5396 treatment.

We investigated whether the protective effects of MHY5396 on FA‐induced kidney fibrosis were associated with modulation of inflammation and lipid metabolism. MHY5396 treatment significantly reduced the expression of inflammation‐related genes (*Ccl2, Ccl5*, and *Cxcl1*) and macrophage markers (*Emr1* and *Cd163*) compared with the FA group (Figure 7A,B). It also inhibited NF‐κB activation, as evidenced by decreased p65 expression and phosphorylation (Figure 7C–E). In situ hybridization showed that inflammatory markers (*Ccl2* and *Emr1*) and fibrosis marker Col1a1 were highly expressed in damaged tubules and macrophage‐infiltrated areas of FA‐treated kidneys, while MHY5396 markedly reduced their expression (Figure 7F–I), indicating its anti‐inflammatory and anti‐fibrotic effects. Additionally, MHY5396 regulated lipid metabolism in renal tubule epithelial cells. It suppressed lipogenesis genes (*Acc, Fasn*, and *Scd1*) while enhancing β‐oxidation (*Acox1* and *Cpt1a*) and mitochondrial function (*Cox4, Cox7b, Atp5g2*, and *mtCytb*) in NRK52E cells (Figure S13). MHY5396 also reduced TG accumulation and restored fatty acid oxidation and OXPHOS protein expression in FA‐induced kidneys (Figures S13 and S14). These findings suggest that MHY5396 alleviates kidney fibrosis by suppressing inflammation and restoring metabolic homeostasis.

### 2.8 MHY5396 Reduces Established Renal Fibrosis Induced by Adenine Diet in Mice

To evaluate the therapeutic effects of MHY5396 on established renal fibrosis, mice were fed an adenine diet (AD, 0.25%) for two weeks, followed by MHY5396 treatment for 1 week (Figure S15A). MHY5396 significantly reduced AD‐induced serum creatinine levels and the expression of kidney injury markers Havcr1 and Lcn2 (Figure S15B,C). Histological analysis showed that MHY5396 alleviated AD‐induced tubule dilation (Figure S15D). MHY5396 also suppressed AD‐induced inflammation, as shown by reduced expression of Il1b, Il6, Ccl2, and lower p65/p‐p65 protein levels (Figure S15E,F). In addition, MHY5396 decreased the expression of the macrophage marker Emr1 and the pro‐fibrotic M2 marker Arg1 (Figure S15G). Fibrosis markers at the gene, protein, and tissue levels were consistently reduced in the MHY5396 group (Figure S15H–J). These results demonstrate the therapeutic anti‐inflammatory and anti‐fibrotic effects of MHY5396 in an established kidney fibrosis model.

### 2.9 Drug Metabolism and Pharmacokinetic Properties of MHY5396

**FIGURE 8:**
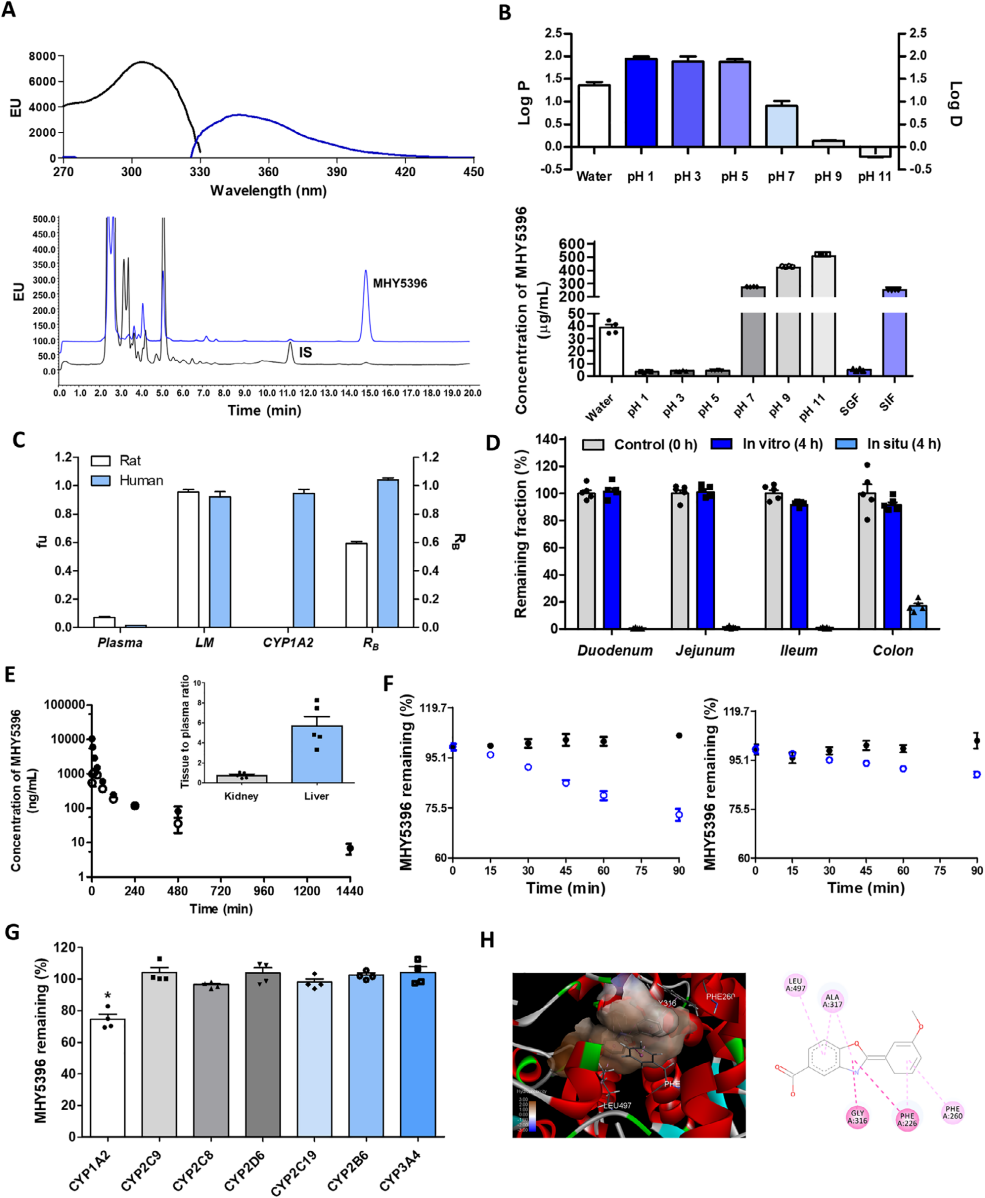
Physicochemical properties and DMPK characteristics of MHY5396. (A) Fluorescence spectra of MHY5396 and representative chromatogram of MHY5396 and IS in rat plasma sample collected 1440 min after intravenous administration in rats. (B) Log P/D and solubility of MHY5396 in phosphate buffers of various pH, SGF, and SIF (*n* = 4). (C) Unbound fractions of biological samples, RB (*n* = 4). (D) Remaining fractions of MHY5396 at 4 h after its injection into the duodenum, jejunum, ileum, and colon loops of the rats (*n* = 5). (E) Plasma concentration versus time profiles of MHY5396 after intravenous (closed circle) and oral (open circle) administration in rats and tissue distribution (tissue to plasma ratio) of MHY5396 at 240 min after intravenous administration at 2 mg/kg in rats, respectively (*n* = 5). (F) Time course of the remaining fractions of MHY5396 in RLM and HLM in the absence (closed circle; without cofactor) and presence (open circle; with cofactor) of NADPH. (G) Relative disappearance of MHY5396 in human recombinant CYP enzymes. (H) Molecular docking analysis demonstrating the binding positions of MHY5396 in human CYP1A2 (PDB code: 2HI4). The bullet symbols and rectangular bars represent the means, while the error bars represent the standard deviations (*n* = 4).

Finally, based on its efficacy in animal disease models, we evaluated drug metabolism and pharmacokinetic properties of MHY5396. MHY5396 displayed a prominent native fluorescence peak intensity at the excitation/emission wavelength pair of 306/348 nm. Isocratic elution using the reversed‐phase column yielded retention times of 14.9 min for MHY5396 and 11.3 min for internal standard (Figure 8A). Intermediate log D values (1.87‒1.94) were observed at acidic pH values (1–5), which were similar to log P values. However, at neutral and basic pH (7‒11), log D values significantly decreased, which aligns with the pH‐dependent solubility of MHY5396 in pH 7.0 SIF (252‒253 µg/mL) and pH 1.2 SGF (4.13‒5.81 µg/mL; Figure 8B). The unbound fractions of MHY5396 in rat plasma, human plasma, rat liver microsomes, human liver microsomes, and CYP1A2 were 0.0683 ± 0.0182, 0.0154 ± 0.0010, 0.954 ± 0.037, 0.922 ± 0.071, and 0.947 ± 0.053, respectively (Figure 8C). The RB of MHY5396 in rats and humans was 0.592 ± 0.029 and 1.04 ± 0.03, respectively (Figure 8C). In vitro isolated loops exhibited remaining fractions of MHY5396 of 87.1%‒109%, whereas in situ loops showed lower values of 0.442%‒23.2% after the same incubation period, and a significant portion was absorbed (Figure 8D; *p* < 0.001). Intravenous administration resulted in a multi‐exponential decline in plasma MHY5396 levels with relatively low plasma clearance (CL; 6.13‒9.30 mL/min/kg), moderate Vss (1058‒1750 mL/kg), and t1/2 of 221‒381 min. Urinary excretion of MHY5396 accounted for only a small fraction (1.26%‒1.65%) of the intravenous dose. Following oral administration, the Cmax of MHY5396 was observed at 15‒60 min. Only minor portions (0.0851%–1.74%) of the dose were recovered as unchanged MHY5396 from the entire gut (Figure 8E and Table S3). Tissue‐to‐plasma concentration ratios (Kp) were 0.744 ± 0.289 and 5.70 ± 2.07 for the kidney and liver, respectively. The remaining fractions of MHY5396 in hepatic microsomes were significantly decreased in the presence of NADPH (with t1/2, LM of 169−252 min for rats and 413−644 min for humans; Figure 8F), and a significant disappearance of MHY5396 was observed only in CYP1A2 (*p* < 0.001; Figure 8G). MHY5396 was docked well within the binding pocket of CYP1A2. Leu497, Ala317, Gly316, Phe226, and Phe260 interacted hydrophobically with MHY5396. Additionally, MHY5396 exhibited a relatively low binding energy of −10.0 kcal/mol at the active site of CYP1A2, indicating a high affinity for the CYP1A2 isoform (Figure 8H). These findings suggested that MHY5396 is characterized by efficient absorption, extensive distribution, and significant hepatic metabolism mediated primarily by CYP1A2. These attributes, combined with the stability of the drug under various conditions, highlight its potential as a therapeutic agent.

## 3 Discussion

The incidence of fibrotic diseases is steadily increasing, and the disease burden caused by fibrosis is substantial [4]. However, there are currently no effective therapies for preventing or reversing fibrosis. The pathogenic mechanisms underlying fibrosis are being actively investigated, leading to the discovery of new therapeutic opportunities. Recent studies have shown that alterations in lipid metabolism are common mechanisms and core pathways central to various fibrotic disorders and are also associated with other metabolic diseases [5, 29, 30]. Genetic modifications or pharmacological targeting of fatty acid metabolic processes have significant potential to inhibit the development of fibrosis. Based on the evidence linking lipid metabolism to fibrosis, drugs targeting lipid metabolism have been actively investigated. In the current study, we focused on the regulation of the nuclear receptors implicated in lipid metabolism, FXR and PPARα. By simultaneously activating these two nuclear receptors, we observed regulatory effects on lipid metabolism and anti‐fibrotic effects in different types of fibrosis models in the liver and kidney. Our results suggest that activating FXR and PPARα possesses significant anti‐fibrotic and metabolic regulatory properties in both liver and kidney fibrosis models, thus highlighting their potential as novel therapeutic targets for fibrotic diseases.

The role of lipid metabolism in the development of fibrotic disorders has been studied extensively. In the liver, changes in lipid metabolism affect pathological fibrosis both directly and indirectly. Under pathological conditions, disruptions in hepatic lipid metabolism can occur, leading to the accumulation of TG in liver cells due to enhanced lipid uptake, excessive lipid synthesis, or impaired lipid breakdown [31]. Fatty liver can be reversed, but if left untreated, long‐term accumulation of lipids can progress to nonalcoholic fatty liver disease and metabolic dysfunction‐associated steatohepatitis (MASH), ultimately leading to liver cirrhosis [32]. The activation of HSCs is primarily influenced by alterations in liver lipid metabolism, highlighting the critical role of lipid metabolism in the development of liver fibrosis. The direct toxic effects of fatty acids represent one of the most extensively studied links between lipid metabolism and fibrosis. Wobser et al. were the first to provide evidence of how lipid accumulation in hepatocytes plays a significant role in the development of fibrosis. Palmitate‐treated hepatocytes induce lipid accumulation and release factors that directly promote liver fibrosis [9]. It has been demonstrated that dual PPAR α/γ agonists regulate lipid metabolism, reducing lipid accumulation in the liver and thereby preventing mitochondrial toxicity, oxidative stress, inflammation, and the progression of fibrosis [33]. Most important studies on the kidney have focused on altered FAO. Using unbiased gene profiling, Kang et al. found that profound alterations in fatty acid metabolism occurred in both animal models of kidney fibrosis and in human subjects with CKD [13]. Using tubular epithelial cells, they showed that impaired FAO leads to ATP depletion, cell death, dedifferentiation, and lipid accumulation—key characteristics of fibrosis. Treatment with a PPARα agonist provided protective effects, suggesting that pharmacological enhancement of FAO can safeguard animals from kidney fibrosis. This link between altered FAO and fibrosis was further confirmed in an age‐related kidney fibrosis model [28]. More recently, Zhou et al. demonstrated that abnormal cannabinoid receptor2/β‐catenin signaling pathway inhibits renal FAO by suppressing PPARα/PGC‐1α axis [34]. In addition, mitochondrial dysfunction is thought to play an important role in the pathogenesis of renal fibrosis [35, 36]. Growing evidence indicates that changes in lipid metabolism are frequently observed in fibrotic diseases. As a result, various preclinical and clinical strategies aimed at reversing these metabolic alterations have emerged as promising approaches for mitigating fibrosis [29].

Activating FXR is an interesting target for treating fibrotic diseases based on its transcriptional activity. The pleiotropic mechanisms involved in the development of liver fibrosis present various therapeutic opportunities, with FXR activation emerging as a well‐established pharmacological target. A range of FXR agonists with diverse physicochemical properties, including bile acid derivatives, non‐steroidal FXR agonists, and partial FXR agonists, are currently undergoing advanced clinical development [37]. The most promising results were obtained from the FLINT trial, which demonstrated the capacity of the FXR agonist, OCA, to improve the histological features of MASH, including hepatic steatosis, hepatocyte ballooning, inflammation, and fibrosis [38]. Based on these results, FXR agonists have been developed to enhance FXR potency and selectivity, while addressing some of the side effects associated with OCA, particularly pruritus and lipid imbalance [39]. Although OCA has shown the potential to improve certain histological features of MASH and liver fibrosis, its development has been hampered by safety concerns, inconsistent efficacy, and regulatory hurdles [40]. Several non‐steroidal FXR agonists with fewer adverse effects are currently being tested in phase II/III clinical trials for the treatment of MASH [41, 42]. In our preclinical tests with MHY5396, we observed similar efficacy in various animal models of liver fibrosis. In addition, no toxicity was observed during 2 months of administration in the animal experiments. The mice did not exhibit an increase in cholesterol or serum AST/ALT levels. Further detailed toxicological studies are needed to comprehensively assess the safety profile of MHY5396, especially considering its prolonged administration and potential effects on other physiological parameters.

In addition to liver fibrosis, FXR agonism is implicated in the development of kidney diseases. FXR activation mitigates renal fibrosis through various mechanisms, including suppression of pro‐inflammatory cytokines, reduction of oxidative stress, and inhibition of EMT in renal tubular cells [24, 43, 44]. It also plays a role in lipid metabolism in the kidneys. Wang et al. demonstrated the protective role of FXR in a diabetic nephropathy (DN) model [45]. FXR deficiency exacerbated kidney injury, whereas the administration of FXR agonists attenuated this injury by reducing proteinuria, glomerulosclerosis, and tubulointerstitial fibrosis. FXR regulated lipid metabolism in the DN model, particularly by decreasing SREBP expression. Furthermore, the FXR/TGR5 dual agonist INT‐767 prevented DN through multiple pathways, including regulation of lipid metabolism and mitochondrial biogenesis [46]. More recently, Xu et al. demonstrated the significant role of FXR in lipid metabolism in a cisplatin‐induced AKI model [47]. Using various genetic models of FXR expression modification, they showed that FXR improves FAO and reduces lipid accumulation via PPARγ in PTECs of the kidney. Our results also indicated the metabolic role of MHY5396 in kidney cells. MHY5396 not only reduced inflammatory and fibrotic responses in animal models but also reduced lipid accumulation and increased mitochondrial components. Together with PPARα agonistic activity, the dual agonist effectively modulated various aspects of kidney fibrosis development, including lipid metabolism.

Through a series of efficacy and pharmacokinetic experiments, we demonstrated the potential of a newly discovered FXR and PPARα dual agonist for the prevention and treatment of fibrotic diseases. We believe that the strength of our study lies in its focus on regulating two different nuclear receptors, which have been extensively studied in metabolic and fibrotic disorders. Since activators for these receptors are already used as medications for these diseases, our approach could facilitate drug development. In addition, we have shown the effectiveness of the drug across the different organs with different mechanisms of disease development. Using major fibrosis models in the liver and kidney, drug treatment significantly reduced the extent of fibrosis. Additionally, changes in lipid metabolism were observed in some models, with noticeable reductions in lipid accumulation. Although the efficacy is evident, there are still limitations of this study. First, our in vivo study primarily focused on preventive measures. While we employed established fibrosis models in both the liver and kidney, these animal models are not fully adequate to demonstrate therapeutic efficacy comprehensively. Further analyses are required to evaluate the compound's potential for clinical translation. Second, the absence of hepatomegaly in response to the dual agonist, despite PPARα activation, represents a limitation of the study and warrants further investigation to determine the molecular basis of this differential physiological outcome. Further studies are needed to elucidate the underlying mechanisms, including the selective regulation of PPARα target genes or potential counteracting effects of FXR activation. Finally, a comprehensive preclinical toxicity assessment is essential to validate its safety for drug development. Both nuclear activators have been reported to cause several adverse effects, especially with prolonged use. Further research is required to fully understand the long‐term implications of these nuclear activators and to develop safer analogs that retain therapeutic efficacy while minimizing toxicity.

We successfully developed novel FXR and PPARα dual agonists with potent anti‐fibrotic and metabolic regulatory effects. Benzoxazole derivatives, particularly MHY5396, emerged as highly effective compounds with low EC50 values. MHY5396 significantly suppressed lipid synthesis and enhanced β‐oxidation in hepatocytes, resulting in reduced lipid accumulation. Furthermore, MHY5396 effectively inhibited the TGFβ‐induced fibrosis response in HSCs and exhibited marked anti‐fibrotic and hepatoprotective effects in mouse models of liver fibrosis. Notably, MHY5396 demonstrated significant anti‐fibrotic and anti‐inflammatory effects in renal cells and mouse models of renal fibrosis. Drug metabolism and pharmacokinetic studies indicated that orally administered MHY5396 was efficiently absorbed and primarily metabolized by hepatic CYP1A2 with negligible urinary excretion. This study highlights the therapeutic potential of MHY5396 as a dual FXR and PPARα agonist. By effectively targeting metabolic dysregulation and fibrosis, MHY5396 offers a novel approach for treating fibrotic diseases, addressing both liver and kidney fibrosis with high efficacy and minimal adverse effects (Figure S16).

## 4 Materials and Methods

### 4.1 Animal Studies

Nine‐week‐old male C57BL/6J mice were obtained from Hyochang Science (Daegu, Republic of Korea) and housed in a laboratory animal facility at the Pusan National University (PNU). All mice were housed under controlled conditions, with a temperature maintained at 23 ± 2°C, a relative humidity of 60 ± 5%, and a 12‐h light/dark cycle. They were provided ad libitum access to food and water.

In the MCD mouse model and HFD‐fed mouse model were used to investigate the effects of MHY5396 on hepatic lipid accumulation and fibrosis. In MCD mouse model, MHY5396 was orally administered daily at doses of 0.4 or 2 mg/kg along with the MCD diet for a duration of 3 weeks. In the HFD‐fed mouse model, MHY5396 was administered orally at a dose of 3 mg/kg daily, together with the HFD (60 kcal% fat), for 8 weeks. To further explore the effects on fibrosis suppression, a thioacetamide (TAA)‐induced liver fibrosis model was used. Liver fibrosis was induced by intraperitoneal injection of TAA at a dose of 150 mg/kg, administered three times a week for 8 weeks. MHY5396 or obeticholic acid (OCA) dissolved in 1% DMSO was given orally at a daily dose of 3 mg/kg. After the treatment period, the mice were euthanized using CO_2_. Serum was collected for biochemical analysis, and liver tissues were immediately harvested and frozen in liquid nitrogen for subsequent quantitative reverse transcription polymerase chain reaction (qRT‐PCR), western blotting, and biological analysis. Furthermore, some liver samples were fixed in 4% paraformaldehyde buffered to neutral pH for histochemical analysis. For long‐term storage, remaining liver samples were stored at −80°C in a deep freezer. To evaluate the therapeutic effects of MHY5396 on liver fibrosis, a TAA‐induced liver fibrosis mouse model was utilized. Liver fibrosis was induced by administering TAA intraperitoneally at a dose of 150 mg/kg three times a week for a duration of 2 weeks. After the induction period, the mice received oral MHY5396 treatment at a dose of 5 mg/kg per day for 1 week. Upon completion of the treatment period, mice were euthanized using CO_2_, and serum and liver tissue samples were harvested for subsequent evaluation.

To assess the potential inhibition of fibrosis in organs other than the liver by MHY5396, we examined its effects on the kidneys. To achieve this, an FA (dissolved in 300 mM NaHCO_3_)‐induced renal fibrosis mouse model was utilized. Renal fibrosis was induced by intraperitoneal injection of 250 mg/kg of FA, followed by daily oral administration of MHY5396 at doses of 1 or 5 mg/kg for 1 week. After the treatment period, the mice were euthanized using CO_2_, and serum and kidney tissues were collected, following the same procedure as used for liver samples. The therapeutic effects of MHY5396 on renal disease were evaluated using an AD‐induced kidney injury model. In this model, renal damage was induced in mice by administering a diet containing 0.25% adenine (TCI; A0149) for 2 weeks. Following the induction period, MHY5396 was administered orally at a dose of 5 mg/kg per day for 1 week, and the mice were maintained on a chow diet. At the end of the treatment period, the mice were euthanized using CO_2_, and serum and kidney tissue samples were collected for further analysis.

### 4.2 Cell Experiments

The HepG2 human hepatocellular carcinoma cell line was obtained from the American Type Culture Collection (ATCC, Manassas, VA). The cells were cultured in Dulbecco's modified Eagle's medium (DMEM) supplemented with 10% (v/v) fetal bovine serum (FBS) and 1% (v/v) penicillin (100 U/mL)–streptomycin (10 µg/mL). Cultures were maintained in a 5% CO_2_‐humidified incubator at 37°C. HepG2 cells were used to assess the dual agonistic effects of the tested compounds, as well as to evaluate the inhibitory effect of MHY5396 on lipid accumulation. The inhibitory effect of MHY5396 on lipid accumulation was additionally evaluated using the AC2F rat liver‐derived cell line under the same conditions as the HepG2 cells. To examine the fibrotic response, LX2 human HSCs were employed. LX2 cells were cultured under the same conditions as HepG2 cells. Following incubation with TGFβ and MHY5396, RNA and protein samples were obtained for further analysis. To assess the effect of MHY5396 on kidney cells, experiments were conducted using NRK52E cells, a rat kidney epithelial cell line, and NRK49F cells, a rat kidney fibroblast cell line, both obtained from ATCC. NRK52E cells were cultured in a medium containing 5% FBS, whereas NRK49F cells were cultured in a medium containing 10% FBS. To evaluate the anti‐inflammatory effect of MHY5396 in kidney cells, NRK52E cells were pretreated with MHY5396 for 30 min, followed by 30 min of treatment with 10 µg/mL lipopolysaccharide (LPS). Additionally, to examine the anti‐fibrotic effect of MHY5396 in kidney cells, the cells were pretreated with MHY5396 for 30 min and then exposed to TGFβ (PEPROTECH, Cranbury, NJ) at a concentration of 10 ng/mL for 24 h. All the cell lines used in this study were authenticated by short tandem repeat analysis to confirm their identity and were confirmed to be mycoplasma free using a mycoplasma detection assay prior to experimentation.

### 4.3 Measurement of Transcriptional Activity

A luciferase assay was conducted to evaluate the transcriptional activities of FXR and PPARα in response to MHYs. HepG2 cells were transfected with the FXR promoter‐FLUC to determine FXR transcriptional activity and with the PPRE‐X3‐TK‐LUC plasmid with PPARα promoter‐FLUC plasmid DNA to determine PPARα transcriptional activity using Lipofectamine 3000 reagent (Invitrogen, Carlsbad, CA). After transfection, the cells were exposed to FXR and PPARα agonists and MHYs. The transcriptional activities of FXR and PPARα were measured using the One‐Glo Luciferase Assay System (Promega, Madison, WI; catalog number E6120). Luminescence was recorded using a luminescence plate reader (Berthold Technologies GmbH & Co., Bad Wildbad, Germany) following the addition of the luciferase substrate. For assessing NF‐κB transcriptional activity in NRK52E cells, the cells were transfected with the NF‐κB promoter‐FLUC plasmid using Lipofectamine 3000 reagent (Invitrogen). After transfection, the cells were pretreated with MHY5396 and then treated with LPS (10 µg/mL) for 2 h. The NF‐κB transcriptional activity was evaluated using the One‐Glo Luciferase Assay System (Promega), and luminescence was recorded following the addition of the luciferase substrate.

### 4.4 Serum Biochemical Measurements

Serum samples collected from mice were incubated at room temperature for 20 min before being centrifuged at 3000 rpm for 20 min to separate blood cells and clotting factors. Serum alanine transaminase (ALT) and aspartate aminotransferase (AST) activities were measured using ALT and AST assay kits (Asan Pharm, Seoul, Republic of Korea) to assess liver injury. Blood urea nitrogen (BUN) levels in the serum were analyzed using a commercial assay kit (SICDIA L‐BUN, Shinyang Diagnostics, Seoul, Republic of Korea; catalog number 1120171) following the manufacturer's guidelines. Creatinine levels in the serum were quantified using the SICDIA CREA assay kit (Shinyang Diagnostics; catalog number 1120051) in accordance with the manufacturer's instructions.

### 4.5 Histological Analysis

Liver and kidney tissues were processed to detect histological changes through the following procedures. The tissues were fixed in 4% paraformaldehyde, and paraffin‐embedded sections were prepared for H&E staining according to standard procedures. To evaluate the extent of hepatic and renal fibrosis as well as tissue damage, Sirius Red (SR) staining was performed using a commercially available kit (Rockville, MD; catalog number VB‐3017). Immunohistochemistry (IHC) was employed to detect specific regions of protein expression in kidney tissues using antibodies targeting antigens. After deparaffinizing and rehydrating paraffin‐embedded kidney sections, the samples were incubated with primary antibodies targeting p‐NF‐κB (Santa Cruz, Santa Cruz, CA; catalog number sc‐136548). The antigen–antibody complexes were visualized using diaminobenzidine (DAB) solution. Hematoxylin counterstaining was used to stain the cell nuclei within the sections. Images were acquired using a microscope (Leam Solution, Seoul, Republic of Korea; catalog number LS30).

### 4.6 Quantification and Statistical Analysis

Differences between two groups were analyzed using Student's *t*‐test, while differences among multiple groups were assessed with analysis of variance (ANOVA). A *p*‐value of < 0.05 was deemed statistically significant. Data analysis was conducted using GraphPad Prism version 5.0 (GraphPad Software Inc., San Diego, CA), and image analysis was performed using ImageJ software (National Institutes of Health, Bethesda, MD). A detailed description of materials and methods can be found in the Supporting Information.

## Author Contributions

**Mi‐Jeong Kim**: data curation, investigation, writing – original and revised draft. **Dong‐Gyun Han**: data curation, investigation. **Hyeon Seo Park**: investigation, resources. **Sugyeong Ha**: investigation. **Sang Gyun Noh**: investigation. **Jeongwon Kim**: investigation. **Ji‐an Yoo**: investigation. **Byeong Moo Kim**: investigation. **Khas Erdene Battogtokh**: resources. **Soohwan Oh**: resources. **Youngmi Jung**: resources. **Youngsuk Jung**: resources. **Hae Young Chung**: resources. **Hyung Ryong Moon**: conceptualization, resources, supervision. **In‐Soo Yoon**: conceptualization, resources, supervision, writing – original and revised draft. **Ki Wung Chung**: conceptualization, supervision, writing – original and revised draft. All authors have read and approved the final manuscript.

## Ethics Statement

All animal experiments conducted in this study adhered to the guidelines for animal experimentation issued by PNU and were reviewed and approved by the Institutional Animal Care Committee of PNU (approval no. PNU‐2024‐0412).

## Conflicts of Interest

The authors declare no conflicts of interest.

## Supporting information




**Supporting Table 1**: Docking score calculated based on Autodock Vina and LeDock simulation.
**Supporting Table 2**. Predicted interaction between the proteins and ligands.
**Supporting Table 3**: Noncompartmental plasma pharmacokinetic parameters of MHY5396 following intravenous and oral administration at 2 mg/kg in rats (n = 5).
**Supporting Table 4**: Validation parameters of HPLC analysis of MHY5396 in rat plasma (n = 5).
**Supporting Table 5**: Primer sequences for qPCR
**Supporting Table 6**: Information of primary antibodies used in Western blotting
**Supporting Figure 1**: Structures of benzoxazole derivatives tested for dual agonist. Structure of MHYs, a dual agonist of the farnesoid X receptor (FXR) and peroxisome proliferator‐activated receptor (PPAR) alpha, based on the benzoxazole scaffold.
**Supporting Figure 2**: Thermal shift assay results of FXR and PPARα ligand‐binding domains (LBDs) in the presence of ligands. (A) Thermal stability of FXR LBD in the absence or presence of OCA (20 µM) or MHY5396 (20 µM). (B) Thermal stability of PPARα LBD in the absence or presence of fenofibric acid (Feno, 20 µM) or MHY5396 (20 µM).
**Supporting Figure 3**: Pharmacophore analysis performed based on interactions between the proteins and ligands. Pharmacophore analysis of (a) obeticholic acid and (b) MHY5396 against FXR, and (c) fenofibrate and (d) MHY5396 against PPARα. Green arrows indicate hydrogen bond (H‐bond) donors, red arrows indicate H‐bond acceptors, and yellow regions indicate hydrophobic interactions or van der Waals forces. The involved amino acid residues are alanine (ALA), arginine (ARG), isoleucine (ILE), leucine (LEU), methionine (MET), phenylalanine (PHE), serine (SER), threonine (THR), tyrosine (TYR), and valine (VAL).
**Supporting Figure 4**: Docking simulation results of MHY5396 on PPARα and FXR using DiffDock‐L. 3D docking structures of the compound MHY5396 with the nuclear receptors PPARα (A) (Peroxisome Proliferator‐Activated Receptor Alpha) and FXR (B) (Farnesoid X Receptor). Polar contacts with MHY5396 highlighted.
**Supporting Figure 5**: Effect of MHY5396 on lipid metabolism in AC2F liver hepatocytes. (A) Schematic diagram of the experiment to confirm genetic changes caused by single treatment with MHY5396 and alterations in OA‐induced lipid metabolism by MHY5396 treatment in AC2F cells. (B) Relative mRNA levels of lipid metabolism‐related genes (*Acc1, Fasn, Dgat2*, and *Scd1*) in MHY5396‐single treated AC2F cells. The results are quantified as a ratio to 18S rRNA. *p < 0.05 compared to the control group. (C) Relative mRNA levels of β‐oxidation‐related genes (*Acox1* and *Cpt1a*) in MHY5396‐single treated AC2F cells. The results are quantified as a ratio to 18S rRNA. *p < 0.05 compared to the control group. (D) Triglyceride (TG) levels in AC2F cells treated with OA, with or without MHY5396. ###P <0.001 compared to the control group. ***p < 0.001 compared to the OA‐treated group. (E) Representative images of Oil red O (ORO) staining of AC2F cells.
**Supporting Figure 6**: Effect of obeticholic acid and fenofibric acid on TGFβ‐induced fibrosis response in LX2 cells and OA‐induced lipid accumulation in hepatocytes. (A) Schematic diagram of the experiment validating the anti‐fibrotic effect of obeticholic acid (OCA) and fenofibric acid in TGFβ‐treated LX2 stellate cells. (B) Relative mRNA levels of fibrosis‐related genes (*Acta2, Col1a2, Col3a1, Fn*, and *Vim*) in TGFβ‐treated LX2 cells with or without OCA treatment. The results are quantified as ratios of *Gapdh*. *p<0.05 compared to the control group. ***p<0.001 compared to the control group. #*p*<0.05 compared to the TGFβ‐ treated group. ##p<0.005 compared to the TGFβ‐treated group. ##*p*<0.005 compared to the TGFβ‐treated group. (C) Relative mRNA levels of fibrosis‐related genes (*Acta2, Col1a2, Col3a1 Fn*, and *Vim*) in TGFβ‐treated LX2 cells with or without fenofibric acid treatment. The results are quantified as ratios of *Gapdh*. ***p<0.001 compared to the control group. #*p*<0.05 compared to the TGFβ‐treated group. ##*p*<0.005 compared to the TGFβ‐treated group. ##*p*<0.005 compared to the TGFβ‐treated group. (D) Schematic diagram of the experiment assessing the effects of OCA and fenofibric acid on oleic acid‐induced lipid accumulation in AC2F cells. (E) Relative triglyceride levels in OA with or without OCA treatment in AC2F cells. ###*p*<0.001 compared to the control group. *p<0.05 compared to the OA‐treated group.(F) Relative triglyceride levels in OA with or without fenofibric acid treatment in AC2F cells. ###*p*<0.001 compared to the control group. ***p<0.001 compared to the OA‐treated group.
**Supporting Figure 7**: Effect of a single oral administration of MHY5396 in mice. (A) MHY5396 (5 mg/kg) was administered orally to mice, which were sacrificed 6 h after treatment. (B) Relative mRNA expression levels of hepatic β‐oxidation–related genes (*Ppara, Acox1*, and *Cpt1a*) in the presence or absence of MHY5396 treatment. Gene expression levels were normalized to 18S rRNA. *p < 0.05 compared to the non‐treated group. (C) Relative mRNA expression levels of lipid synthesis–related genes (*Acca, Scd1, Lpin1*, and *Chrebp*) with or without MHY5396 treatment. Gene expression levels were normalized to 18S rRNA. *p <0.05 compared to the non‐treated group.
**Supporting Figure 8**: Effect of MHY5396 on MCD‐induced body weight changes, histological and fibrotic changes in liver. (A) Final mouse body weight on the last day of the experiment. (B) Serum ALT levels in MCD diet mice with or without MHY5396 treatment.***P<0.001 compared to the control mouse group. #P<0.05 compared to the MCD diet mouse group. (C) Representative hematoxylin & eosin (H&E) staining of mouse liver tissue sections.(D) Relative mRNA levels of hepatic lipid metabolism‐related genes (*Srebp1, Srebp2, Pparr. Acca. Fasn*, and *Scd1*) with or without MHY5396 treatment. The results are quantified as ratios of 18S rRNA. *P<0.05 compared to the MCD diet mouse group. (E) Relative mRNA levels of hepatic fibrosis‐related genes (*Tgfb1, Acta2, Fn*, and *Col1a1*) with or without MHY5396 treatment. The results are quantified as ratios 18S rRNA. *P<0.05 compared to the MCD diet mouse group. (F) Representative in situ hybridization (ISH) images detected with a Vimentin (*Vim*, red) probe in methionine choline deficient (MCD) diet‐induced mouse liver with or without MHY5396 treatment (Scale bar = 100 µm).
**Supporting Figure 9**: Effect of MHY5396 on Pcna, Krt23, and Ki67 gene expression in liver. (A) Relative mRNA levels of Pcna following MHY5396 administration. The results are quantified as a ratio to 18S rRNA. (B) Relative mRNA levels of Krt23 following MHY5396 administration. The results are quantified as a ratio to 18S rRNA. (C) Relative mRNA levels of Ki67 following MHY5396 administration. The results are quantified as a ratio to 18S rRNA.
**Supporting Figure 10**: Effect of MHY5396 on high‐fat diet (HFD)‐induced body weight changes and fatty liver development. (A) Experimental scheme illustrating HFD‐induced fatty liver and the effect of MHY5396 (n = 6). (B) Final mouse body weight at the end of the experiment. **p < 0.005 compared to the control group. (C) Hepatic triglyceride (TG) levels in the livers of HFD‐fed mice with or without MHY5396 treatment. ***p < 0.001 compared to the control group; #*p* < 0.05, ##*p* < 0.005 compared to the HFD group. (D) Representative Oil Red O (ORO) staining of mouse liver sections. (E) Relative mRNA expression levels of hepatic lipid metabolism‐related genes (*Acca, Fasn, Scd1*, and *Ppara*) with or without MHY5396 treatment. Results are normalized to 18S rRNA. #*p* < 0.05 compared to the HFD group.
**Supporting Figure 11**: MHY5396 alleviates thioacetamide (TAA)‐induced liver fibrosis in mice when administered post‐treatment. (A) Experimental scheme to assess the therapeutic effect of MHY5396 in TAA‐induced established liver fibrosis model (n = 7‐10). (B) Representative H&E staining of mouse liver tissue sections. (C) Serum AST levels in TAA‐treated mice with or without MHY5396 treatment. **p<0.05 compared to the control mouse group. #P<0.05 compared to the TAA‐treated group. (D) Relative mRNA levels of hepatic fibrosis‐related genes (*Acta2, Col1a2, Col3a1, Tgfb*, and *Vim*) with or without MHY5396 treatment. The results are quantified as ratios of *Gapdh*. **p<0.005, ***p<0.001 compared to the control mouse group. #*p*<0.05 compared to the TAA‐injected mouse group. (E) Col1a1 and Vimentin protein levels were determined in TAA‐injected mouse livers with or without MHY5396 treatment. α‐tubulin was used as the loading control. Relative protein expressions were quantified using densitometry. ***p<0.001 compared to the control group. #*p*<0.05, ###*p*<0.001 compared to the TAA‐treated group. (F) Representative SR staining of mouse liver tissue sections. Positive areas for SR staining were calculated to determine the extent of hepatic fibrosis in each experimental group. ***p<0.001 compared to the control mouse group. ###*p*<0.001 compared to the TAA‐injected mouse group.
**Supporting Figure 12**: Effect of MHY5396 on cell viability of NRK52E cells and TGFβ‐induced Smad signaling changes in NRK49F cells. (A) The cytotoxicity of MHY5396 was evaluated in a dose‐dependent manner in NRK52E cells. Results were expressed as percentages. (B) Protein levels of Smad signaling proteins (such as Smad2/3, pSmad2, and pSmad3) were determined in NRK49F cells treated with TGFβ, with or without MHY5396 treatment. GAPDH was used as a loading control. Relative protein expressions were quantified using densitometry. **p<0.005, ***p<0.001 compared to the control group.
**Supporting Figure 13**: MHY5396 modulates lipid metabolism in NRK52E renal epithelial cells. (A) Schematic diagram of the experiment to confirm genetic changes caused by single treatment with MHY5396 and alterations in oleic acid (OA)‐induced lipid metabolism by MHY5396 treatment in NRK52E cells. (B) Relative mRNA levels of lipid metabolism‐related genes (*Acc1, Fasn*, and *Scd1*) in MHY5396‐single treated NRK52E cells. The results are quantified as a ratio to 18S rRNA. *p < 0.05 compared to the control group. (C) Relative mRNA levels of β‐oxidation‐related genes (*Acox1* and *Cpt1a*) in MHY5396‐single treated NRK52E cells. The results are quantified as a ratio to 18S rRNA. *p < 0.05 compared to the control group. (D) Relative mRNA levels of mitochondria‐related genes (such as Cox4, Cox7b, Atp5g2, and mtCytb) in MHY5396‐single treated NRK52E cells. The results are quantified as a ratio to 18S rRNA. * p < 0.05 compared to the control group. (E) Triglyceride (TG) levels in NRK52E cells treated with OA, with or without MHY5396. ###p < 0.001 compared to the control group. **p < 0.005 compared to the OA‐treated group. (F) Representative images of Oil red O (ORO) staining of NRK52E cells.
**Supporting Figure 14**: MHY5396 decreases lipid accumulation induced by FA‐treatment and increases OXPHOS, β‐oxidation‐related proteins in kidne. (A) Triglyceride (TG) levels in folic acid (FA)‐induced mouse kidneys with or without MHY5396 treatment. The results are quantified as a ratio to kidney weight. ###p < 0.001 compared to the control group. **p < 0.005, ***p < 0.001 compared to the FA‐treated group. (B) Protein levels of β‐oxidation‐related proteins (OXPHOS, *Acox1*, and *Cpt1α*) were determined in FA‐induced mouse kidneys with or without MHY5396 treatment. α‐tubulin was used as a loading control. Relative protein expressions were quantified using densitometry. *p<0.05, **p<0.005 compared to the control group. #p<0.05 compared to the FA‐treated group.
**Supporting Figure 15**: MHY5396 mitigates established renal fibrosis induced by an adenine diet (AD) in mice. (A) Experimental scheme to assess the therapeutic effect of MHY5396 in AD‐induced established renal fibrosis model (n = 4‐5). (B) Serum creatinine levels in AD‐fed mice with or without MHY5396 treatment. **p<0.005 compared to the control mouse group. #p<0.05 compared to the AD‐treated mouse group. (C) Relative mRNA levels of kidney damage‐related genes (*Havcr1, Spp1*) in AD‐fed mouse kidneys with or without MHY5396 treatment. ***p<0.001 compared to the control mouse group. #p<0.05 compared to the AD‐fed mouse group. (D) Representative H&E staining of mouse kidney tissue sections. (E) Relative mRNA levels of kidney inflammation‐related genes (*Il1b, Il6, and Ccl2*) in AD‐fed mouse kidneys with or without MHY5396 treatment. ***p<0.001 compared to the control mouse group. #p<0.05 compared to the AD‐fed mouse group. (F) Protein levels of p65 and p‐p65 in AD‐fed mouse kidneys with or without MHY5396 treatment. α‐tubulin was used as the loading control. Relative protein expressions were quantified using densitometry. *p<0.05 compared to the control group. #p<0.05 compared to the AD‐fed group. (G) Relative mRNA levels of macrophage marker genes (*Emr1, Arg1*) in AD‐fed mouse kidneys with or without MHY5396 treatment. ***p<0.001 compared to the control mouse group. #P<0.05 compared to the AD‐fed mouse group. (H) Relative mRNA levels of fibrosis‐related genes (*Col1a2, Tgfb*) in AD‐fed mouse kidneys with or without MHY5396 treatment. ***p<0.001 compared to the control mouse group. #p<0.05 compared to the AD‐fed mouse group. (I) Protein levels of COL1 and VIM in AD‐fed mouse kidneys with or without MHY5396 treatment. α‐tubulin was used as the loading control. Relative protein expressions were quantified using densitometry. *p<0.05, ***p<0.001 compared to the control group. #p<0.05 compared to the AD‐fed group. (J) Representative images of SR staining of mouse kidney tissue sections.
**Supporting Figure 16**: Benzoxazole derivatives as potent FXR and PPAR alpha dual agonists with anti‐fibrosis and metabolic regulatory effect.

## Data Availability

The data, analytical methods, and study materials will be made available to other researchers for purposes of replicating the procedure and are available by contacting the corresponding authors.
