# Supplementary material for: Benzoxazole Derivatives as Potent FXR and PPARα Dual Agonists With Anti‐Fibrotic and Metabolic Regulatory Effects
Source: MedComm (2020). 2025 Oct 26;6(11):e70442. doi: 10.1002/mco2.70442 (PMC12554789; doi:10.1002/mco2.70442)
Supplement: Supplementary file 1 — Supporting Table 1: Docking score calculated based on Autodock Vina and LeDock simulation. Supporting Table 2. Predicted interaction between the proteins and ligands. Supporting Table 3: Noncompartmental plasma pharmacokinetic parameters of MHY5396 following intravenous and oral administration at 2 mg/kg in rats (n = 5). Supporting Table 4: Validation parameters of HPLC analysis of MHY5396 in rat plasma (n = 5). Supporting Table 5: Primer sequences for qPCR Supporting Table 6: Information of primary antibodies used in Western blotting Supporting Figure 1: Structures of benzoxazole derivatives tested for dual agonist. Structure of MHYs, a dual agonist of the farnesoid X receptor (FXR) and peroxisome proliferator‐activated receptor (PPAR) alpha, based on the benzoxazole scaffold. Supporting Figure 2: Thermal shift assay results of FXR and PPARα ligand‐binding domains (LBDs) in the presence of ligands. (A) Thermal stability of FXR LBD in the absence or presence of OCA (20 µM) or MHY5396 (20 µM). (B) Thermal stability of PPARα LBD in the absence or presence of fenofibric acid (Feno, 20 µM) or MHY5396 (20 µM). Supporting Figure 3: Pharmacophore analysis performed based on interactions between the proteins and ligands. Pharmacophore analysis of (a) obeticholic acid and (b) MHY5396 against FXR, and (c) fenofibrate and (d) MHY5396 against PPARα. Green arrows indicate hydrogen bond (H‐bond) donors, red arrows indicate H‐bond acceptors, and yellow regions indicate hydrophobic interactions or van der Waals forces. The involved amino acid residues are alanine (ALA), arginine (ARG), isoleucine (ILE), leucine (LEU), methionine (MET), phenylalanine (PHE), serine (SER), threonine (THR), tyrosine (TYR), and valine (VAL). Supporting Figure 4: Docking simulation results of MHY5396 on PPARα and FXR using DiffDock‐L. 3D docking structures of the compound MHY5396 with the nuclear receptors PPARα (A) (Peroxisome Proliferator‐Activated Receptor Alpha) and FXR (B) (Farnesoid X Receptor). Po [file MCO2-6-e70442-s001.pdf]

Supporting data

Benzoxazole derivatives as potent FXR and PPARα dual agonists with anti-fibrotic and metabolic regulatory effects

Mi-Jeong Kim[a,#], Dong-Gyun Han[b,#], Hyeon Seo Park[b], Sugyeong Ha[a], Sang Gyun Noh[a], Jeongwon Kim[a], Ji-an Yoo[a], Byeong Moo Kim[a], Battogtokh Khas Erdene[c], Soohwan Oh[c], Youngmi Jung[d], Youngsuk Jung[a], Hae Young Chung[a], Hyung Ryong Moon[b,*], In-Soo Yoon[b,*], Ki Wung Chung[a,*]

[a]*Department of Pharmacy and Research Institute for Drug Development, College of Pharmacy, Pusan National University, Busan 46241, Republic of Korea*

[b]*Department of Manufacturing Pharmacy and Research Institute for Drug Development, College of Pharmacy, Pusan National University, Busan 46241, Republic of Korea*

[c]*College of Pharmacy, Korea University, 2511 Sejong-ro, Sejong, 30019, Republic of Korea*

[d]*Department of Biological Sciences, College of Natural Science, Pusan National University, Pusan 46241, Republic of Korea*

[#]*These authors contributed equally*

*Correpsonding authors

Hyung Ryong Moon

E-mail: mhr108@pusan.ac.kr

In-Soo Yoon

E-mail: insoo.yoon@pusan.ac.kr

Ki Wung Chung

E-mail: kieungc@pusan.ac.kr

Materials and methods

1. Cell viability, proliferation and cytotoxicity assay

The cytotoxicity of MHY5396 was assessed using the Ez-Cytox reagent kit (DoGen Bio, Seoul, Republic of Korea; catalog number EZ-3000). All procedures were performed according to the manufacturer's instructions. In brief, cells were plated into 96-well plates and treated with different concentrations of MHY5396. After a 24-hour incubation at 37 °C in a 5% CO_2 humidified incubator, Ez-Cytox reagent was added, and the absorbance was measured to determine cytotoxicity.

2. Docking simulation

The crystal structures of FXR and PPARα were sourced from the RCSB PDB database, specifically PDB IDs 4OIV for FXR and 1K71 for PPARα (https://www.rcsb.org/; accessed on May 30, 2024). The 3D structure of MHY5396 was designed using ChemSketch (ACD/Labs, Toronto, ON, Canada) and the positive controls, OCA (for FXR) and fenofibric acid (for PPARα), were obtained from the PubChem database (https://pubchem.ncbi.nlm.nih.gov/; accessed on May 30, 2024). Docking simulations were performed using two programs, AutoDock Vina 1.1.2 (Scripps Research, San Diego, CA) and LeDock (Lephar, http://www.lephar.com). Preparation for docking included adding hydrogen atoms and assigning charges to the compounds using UCSF Chimera (UCSF, San Francisco, CA). Pharmacophore analysis was performed using LigandScout 4.3 (Inte:Ligand, Vienna, Austria) to investigate potential interactions between the receptors and ligands. Additional docking simulations were performed with DiffDock-L (https://github.com/gcorso/DiffDock) for independent benchmark tests.

3. Thermal shift assay

Thermal shift assays were conducted in clear 96-well plates (Bio-Rad) using SYPRO Orange dye (Invitrogen) as the fluorescent reporter and the ligand-binding domains (LBDs) of nuclear receptors as target proteins. FXR LBD (SRP2137, Sigma) and PPARα LBD (SRP2054, Sigma) were tested with specific ligands in separate experiments. For the FXR LBD assay, 12 μL of test compound solution (final concentrations: OCA, 20 μM; MHY5396,

20 μM) prepared in assay buffer (10 mM Tris-HCl, pH 8.3; 5 mM DTT; 0.5 mM EDTA; 100 mM NaCl) was mixed with 6 μL of FXR LBD (final protein concentration: 5 μM) and 2 μL of SYPRO Orange (10× final concentration). For the PPARα LBD assay, 4 μL of test compound solution (final concentrations: fenofibric acid, 20 μM; MHY5396, 20 μM) in the same assay buffer was combined with 14 μL of PPARα LBD (final protein concentration: 10 μM) and 2 μL of SYPRO Orange. Fluorescence changes due to temperature-dependent protein unfolding were monitored in duplicate using an iCycler system (Bio-Rad), with a temperature gradient from 20 °C to 90 °C at a ramp rate of 0.5 °C per minute. Fluorescence was recorded at 300 nm excitation and 570 nm emission. Melting temperatures (Tm) were determined by calculating the first derivative of the fluorescence curves using GraphPad Prism 5 software.

4. TG assay

To measure TG levels in the liver tissue and cells, the tissue and cells were homogenized in a 5% nonyl phenoxypolyethoxylethanol (NP-40) detergent solution. The tissue was heated to 90 ℃, allowed to cool to room temperature, and this process was repeated, followed by centrifugation to obtain the supernatant. To measure TG levels, we used a commercially available assay kit from BIOMAX (Guri, Republic of Korea; catalog number PicoSens^{TM} Triglyceride Assay Kit, BM-TGR-100) following the manufacturer's instructions. TG levels were quantified relative to the protein levels.

5. RNA extraction and qRT-PCR

Gene expression in tissue and cell samples was assessed using qRT-PCR. Total RNA was extracted from the samples with TRIzol reagent (Invitrogen, Carlsbad, CA). To extract RNA, liver and kidney tissues, as well as cells, were homogenized in TRIzol reagent. Subsequently, 200 μL of chloroform was added to the homogenized sample (1 mL), followed by vigorous shaking and a 15-minutes incubation. After centrifugation at 13,000 rpm for 15 minutes at 4 ℃, the aqueous phase (supernatant) was carefully transferred to fresh tubes. An equal volume of isopropanol was added to the supernatant, and the samples were incubated for 15 minutes before being centrifuged at 13,000 rpm for 15 minutes at 4 ℃. The supernatants were discarded, and the RNA pellets were washed once with 75% ethyl alcohol.

Subsequently, the pellets were air-dried and dissolved in diethyl pyrocarbonate (DEPC)-treated water, followed by a 10-minutes incubation at 56 °C. The isolated RNA was reverse transcription using a cDNA synthesis kit (GenDEPOT, Katy, TX). qRT-PCR was performed using SYBR Green Master Mix (BIOLINE, Taunton, MA) and the CFX Connect system (Bio-Rad, Hercules, CA). Primer sequences for mouse, rat, and human genes were designed using Primer3Plus software and are provided in Table S1. For the qRT-PCR data analysis, the $2^{-\Delta\Delta CT}$ method was employed for relative quantification. This method calculates the fold change in gene expression relative to a control sample, normalized to an internal reference gene.

6. Protein extraction

Protein extraction was performed within a controlled temperature range of 0–4 °C to preserve protein stability. Various methods can be employed to extract proteins from tissues and cells. Specifically, for liver and kidney tissues, protein extraction was performed using ProEX™ CETi protein extract solution (Translab, Daejeon, Republic of Korea) supplemented with protease inhibitor cocktails (Xpert Protease Inhibitor Cocktail Solution [100X], GenDEPOT) and a phosphate inhibitor, following the manufacturer's protocols. These supplements were incorporated to mitigate protein degradation and dephosphorylation. For total cellular protein extraction, RIPA buffer (Cell Signaling Technology, Danvers, MA) supplemented with a protease inhibitor cocktail was used. To obtain cytoplasmic and nuclear extracts, NE-PER Nuclear and Cytoplasmic Extraction Reagents (Thermo Fisher Scientific, Waltham, Massachusetts; Catalog number 78833) were employed, adhering to the manufacturer's instructions. Protein concentrations were determined using the Pierce™ BCA Protein Assay Kit (Thermo Fisher Scientific, catalog number 23227) for accurate quantification.

7. Western blotting analysis

The extracted proteins (5-20 μg) were combined with 4X sample buffer (Bio-Rad; catalog number 1610747) and heated to 100 °C for 5 minutes to denature the proteins. Equal amounts of protein samples were resolved on 6%-13% sodium dodecyl sulfate-polyacrylamide gel (SDS-PAGE) electrophoresis and subsequently electrophoretically

transferred onto polyvinylidene fluoride (PVDF) membranes (GVS, Sanford, ME) using the Bio-Rad Western Blotting system (Philadelphia, PA). The membranes were incubated with either 5% skim milk or 3% bovine serum albumin (BSA) in blocking buffer for 2 hours to block nonspecific binding. After blocking, the membranes were washed three times for 10 minutes each with Tris-buffered saline containing 0.1% Tween 20 (PBS-Tween 20; 50 mM Tris; 137 mM NaCl, pH 7.4). The membranes were then incubated overnight at 4 ℃ with specific primary antibodies diluted to concentrations ranging from 1:500 to 1:2,000, as listed in Table S2. The following day, the membranes were washed with TBS-Tween 20 for 10 minutes and incubated for 1 hour at room temperature with horseradish peroxidase (HRP)-conjugated anti-mouse or anti-rabbit secondary antibodies (GeneTex, Irvine, CA; catalog numbers GTX213111-01 and GTX213110-01) diluted 1:10,000. Immunoblot signal detection was performed using Western Bright Peroxide Solution (Advansta, San Jose, CA) and visualized with the Bio-Rad ChemiDoc imaging system (Bio-Rad), in accordance with the manufacturer's instructions. Each Western blot experiment was conducted at least three times to ensure the reliability of the results.

8. Histological analysis

Liver and kidney tissues were processed to detect histological changes through the following procedures. The tissues were fixed in 4% paraformaldehyde, and paraffin-embedded sections were prepared for hematoxylin and eosin (H&E) staining according to standard procedures. To evaluate the extent of hepatic and renal fibrosis as well as tissue damage, Sirius Red (SR) staining was performed using a commercially available kit (Rockville, MD; catalog number VB-3017). Immunohistochemistry (IHC) was employed to detect specific regions of protein expression in kidney tissues using antibodies targeting antigens. After deparaffinizing and rehydrating paraffin-embedded kidney sections, the samples were incubated with primary antibodies targeting p-NF-kB (Santa Cruz, Santa Cruz, CA; catalog number sc-136548). The antigen-antibody complexes were visualized using diaminobenzidine (DAB) solution. Hematoxylin counterstaining was used to stain the cell nuclei within the sections. Images were acquired using a microscope (Leam Solution, Seoul, Republic of Korea; catalog number LS30).

9. Oil red O (ORO) staining

To perform ORO staining on tissue sections, frozen sections were prepared using an optimal cutting temperature (OCT) compound to prevent lipid loss. The tissues were briefly fixed with 4% paraformaldehyde and dehydrated in 30% sucrose for 24 hours. Following dehydration, the tissues were embedded in OCT compound and frozen at -80 °C. After removing the OCT compound by soaking the tissue sections in distilled water, ORO staining was performed. The cells were washed with PBS, fixed with 4% formaldehyde for 10 minutes, and subjected to ORO staining. The tissues and cells were incubated with 60% isopropyl alcohol for 10 minutes. The tissues were then treated with an ORO solution (prepared by diluting ORO in isopropyl alcohol and distilled water in a 3:2 ratio; Sigma Aldrich, Saint Louis, MO) at 60 °C for approximately 10 minutes, while the cells were incubated with the ORO solution at room temperature for 1 hour. After washing with 60% isopropyl alcohol and distilled water, hematoxylin staining and mounting were performed. Images were captured using a microscope (LS30).

10. In situ hybridization (ISH)

ISH was performed on formalin-fixed, paraffin-embedded tissue samples using the RNAscope 2.5 HD Red, RNAscope 2.5 HD Brown, or RNAscope 2.5 HD Duplex Detection kits (Biotechne, Minneapolis, MN; catalog numbers 322360, 322310, and 322500), following the manufacturer’s guidelines. Specific probes targeting RNA molecules within the tissue were utilized for assay. The probes used included Mm-Col1a1 (cat# 319371), Mm-Ccl2 (cat# 311791), Mm-Emr1 (cat# 317961-C2) and Mm-Vim (cat# 457961). The liver and kidney tissue samples were subjected to hybridization with the corresponding probes to ensure specific binding to complementary RNA sequences in the tissue sections. After hybridization, the sections were processed according to the detection system provided with the RNAscope assay kits. Images of the stained sections were captured using a microscope (LS30).

11. Immunofluorescence

The cells were fixed in 4% paraformaldehyde for 10 minutes and washed three times with ice-cold PBS. Permeabilization was performed with 0.25% Triton X-100 in PBS for 10

minutes. To minimize nonspecific antibody binding, the cells were blocked with 1% BSA and 0.1% Tween 20 in PBS for 30 minutes at room temperature. Cells were then incubated overnight at 4 °C with the primary antibodies, αSMA (Santa Cruz; catalog number sc-32251) or NF-κB (Santa Cruz; catalog number sc-514451), diluted 1:100 in blocking buffer. Following PBS washes to remove unbound antibodies, cells were incubated with a secondary antibody (goat anti-mouse IgG (H+L) cross-absorbed secondary antibody, Alexa Fluor™ 488, Invitrogen, Waltham, Massachusetts; catalog number A11001) for 1 hour at room temperature in the dark. The fluorescently labeled secondary antibody specifically bound to the primary antibody, enabling visualization of the target protein. To stain nuclei, cells were counterstained with 4,6-diamidino-2-phenylindole (DAPI) in PBS for 5 minutes. Images of the stained cells were captured using a fluorescence microscope (LS30).

12. Materials and animals for pharmacokinetic studies

β-nicotinamide adenine dinucleotide phosphate (NADPH) and phosphate-buffered saline (PBS), were purchased from Sigma-Aldrich, Inc. (St. Louis, MO, USA). Ertugliflozin (purity > 99%), utilized as an internal standard (IS), was purchased from MedKoo Bioscience, Inc. (Morrisville, NC, USA). Octanol, acetonitrile (ACN) and methanol (MeOH) of analytical grade were purchased from Honeywell, Inc. (Muskegon, MA, USA). Pooled hepatic microsomes from male Sprague-Dawley rats (RLM) and male humans (HLM) were purchased from Discovery Life Sciences (Los Osos, CA, USA). Recombinant CYP enzymes (CYP1A2, CYP2B6, CYP2C8, CYP2C9, CYP2D6, and CYP3A4) were obtained from SPMED (Busan, Korea). The protocol for the present rat study was approved by the Institutional Animal Care and Use Committee of Pusan National University on July 11, 2023 (approval number: PNU-2023-0340).

13. Quantitative bioanalysis of MHY5396 and method validation

A 100 μL biological sample underwent protein removal using 300 μL of ACN containing 500 ng/mL IS, followed by vortex-mixing for 3 min and centrifugation at 15,000 × g for 10 min at 4°C. Subsequently, 320 μL of supernatant was collected, dried under vacuum using a SpeedVac (Eyela, Tokyo, Japan), and reconstituted with 50 μL of mobile phase before injection into a high-performance liquid chromatography-fluorescence detector (HPLC-FLD) system. The fluorescence detector (2475 FLR Detector; Waters Co., Milford, MA, USA) was

connected to an HPLC (ACQUITY Arc LC System; Waters Co.). The optimal excitation and emission wavelengths for MHY5396 were determined through fluorescence spectrum analysis (in methanol at 1000 ng/mL). The stationary phase consisted of a Sunfire C18 column (250 × 2.1 mm, 4.6 μm, 100 Å, Waters Co.) protected by a C18 guard column (SecurityGuard HPLC Cartridge System, Phenomenex, Torrance, CA, USA). The isocratic mobile phase comprised ACN, MeOH, and 0.1% trifluoroacetic acid in deionized water (DIW) (55:25:20, v/v/v). The pump flow rate and column oven temperature were set at 1.0 mL/min and 40°C, respectively. The injection volume and total run time were 20 μL and 20 min, respectively. Excitation and emission wavelengths for MHY5396 were set at 306 and 348 nm, respectively, and for IS at 277 and 320 nm. Stock solutions of MHY5396 and IS were prepared in MeOH at 1 mg/mL and diluted to generate working standard concentrations ranging from 0.1 to 50 μg/mL for MHY5396, and 50 μg/mL for IS. Calibration standards were prepared by spiking each working standard solution into blank rat plasma to achieve final concentrations of 1, 2, 5, 10, 20, 50, 100, 200, and 500 ng/mL. The quality control (QC) concentrations for scopoletin were established as follows: 1 (lower limit of quantification, LLOQ), 3 (low quality control, LQC), 150 (medium quality control, MQC), and 450 ng/mL (high quality control, HQC). This HPLC-FLD method underwent validation studies according to the FDA guidelines for bioanalytical method validation, addressing selectivity, linearity, sensitivity, reproducibility, and stability. Detailed information on the validation process is provided in the Supplementary Information.

14. In vitro physicochemical characterization of MHY5396

The physicochemical properties of MHY5396, including lipophilicity, solubility, protein binding, and blood distribution, were evaluated using relevant in vitro techniques. Simulated gastric fluid (SGF) consisted of 0.2% (w/v) sodium chloride, 0.32% (w/v) pepsin, and 0.7% (v/v) HCl (final pH = 1.2). Simulated intestinal fluid (SIF) was prepared by dissolving 3 mM sodium taurocholate and 0.1% (w/v) pancreatin in phosphate buffer (final pH = 7.0). The partition coefficient of MHY5396 between aqueous phases (e.g., DIW at various pH levels or phosphate buffer) and octanol was determined at 25 ± 0.5°C, with both phases pre-saturated. MHY5396 (below the solubility limit) was dissolved in pre-saturated octanol, mixed in equal volumes with pre-saturated aqueous phase, incubated in a shaking incubator for 24 h, and then centrifuged at 15,000 × g for 10 min. The concentration of MHY5396 in octanol and aqueous samples was quantified using the HPLC-FLD method. The

solubility of MHY5396 was evaluated by adding an excess of the compound to glass vials containing 0.2 mL DIW, phosphate buffer at various pH levels (1.0, 3.0, 5.0, 7.0, 9.0, and 11.0), SGF, and SIF, followed by incubation in a shaking incubator at 500 rpm, 37°C for 48 h, and centrifugation at 15,000 × g for 10 min. The supernatant was filtered through a 0.20 μm syringe filter to remove insoluble solids, and the concentration of MHY5396 in the filtrate was measured using the HPLC-FLD method. Furthermore, the unbound fraction in various biological samples (plasma, RLM, HLM, and CYP1A2) and the blood-to-plasma concentration ratio (R_B) of MHY5396 were measured to assess plasma protein binding and blood distribution, as detailed in the Supplementary Information.

15. In situ closed-loop and in vivo pharmacokinetics studies in rats

In vivo closed-loop studies were conducted on rats following established procedures, with additional details provided in the Supplementary Information. Fasted rats underwent femoral artery and vein catheterization and were anesthetized with an intramuscular injection of Zoletil 20 mg/kg as previously described[1]. Rats received either a single intravenous solution (2 mg/kg; dissolved in a vehicle composed of DMSO, ethanol, PEG400, and saline in a ratio of 2:5:33:60, v/v/v/v) or an oral suspension (2 mg/kg; suspended in 0.5% methylcellulose) of MHY5396. Blood samples (100–250 μL) were collected from the femoral artery at specific time points for both intravenous and oral administration. Plasma was obtained by centrifuging the blood samples at 6,000 × g for 5 min at 4°C. Urine was collected over 24 h, and the entire gastrointestinal content (including feces) was extracted with methanol according to established protocols. Plasma, urine (Ae_U), and gastrointestinal (Ae_G) samples were analyzed using the HPLC-FLD method. The pharmacokinetic data in vivo were analyzed using traditional non-compartmental analysis alongside appropriate pharmacokinetic parameters determined using WinNonlin® Ver. 3.1 software (NCA200 and 201; Certara, Inc., Princeton, NJ, USA). Additionally, rats were administered MHY5396 intravenously through the femoral vein at a dose of 2 mg/kg. 240 min post-administration, the liver and kidneys were separated, washed with saline, dried with clean paper, and their weights were measured. Each tissue was homogenized in ice-cold saline using the FastPrep-24™ 5G bead beating system (MP Biomedicals, OH, USA). The homogenates of the liver and kidneys were then analyzed using the HPLC-FLD method.

16. In vitro metabolic study using liver microsomes and recombinant enzymes

Microsomes (with a protein concentration of 1 mg/mL from either RLM or HLM) or human recombinant cytochrome P450 (CYP) isoforms (for CYP1A2, 2C9, 2C8, 2D6, 2B6, and 3A4 at 50 pmol/mL) were used with cofactors (1 mM NADPH in 100 mM pH 7.4 phosphate buffer) for Phase I reactions. MHY5396 (5 μM) was added to initiate the metabolic reaction. The mixture was incubated at 37°C, 500 rpm for 0, 15, 30, 45, 60, and 90 min in a thermomixer (ThermoMixer C, Eppendorf, Hamburg, Germany), after which 50 μL of the reaction mixture was removed and immediately transferred to another container. To terminate the metabolic reaction, 100 μL of cold acetonitrile containing IS was added to the microtube. The concentration of MHY5396 in the reaction mixture samples was determined using the HPLC-FLD method. The intrinsic clearance of MHY5396 in microsomes ($CL_{int,LM}$) was evaluated using the in vitro half-life ($t_{1/2,LM}$) method. $t_{1/2,LM}$ was calculated from the slope of the drug residual profile, which represents the log percentage against incubation time, and $CL_{int,LM}$ (mL/min/mg protein) was determined accordingly.

$$CL_{int,LM} = \frac{0.693}{t_{1/2}} \cdot \frac{\text{mL incubation}}{\text{mg LM}} \cdot \frac{1}{fu_{LM}} \quad (1)$$

The $CL_{int,LM}$ can be scaled up to its corresponding whole-organ level by physiological scaling factors (134 and 40 mg LM/g organ for rat liver and human liver, respectively)[2,3].

17. Molecular docking study

A molecular docking investigation was conducted to explore the interaction between the ligand (MHY5396) and the receptor (human CYP1A2), based on enzyme metabolism findings. X-ray crystallographic data for human CYP1A2 (PDB code: 2HI4) was retrieved from the Research Collaboratory for Structural Bioinformatics (RCSB) Protein Data Bank (https://www.rcsb.org/)[4]. The MHY5396 structure was generated and energy-minimized using Chem3D Pro 12.0 (Cambridge Soft Co., Cambridge, MA, USA). Molecular docking studies were carried out using AutoDock Vina 1.1.2 software (Molecular Graphics Laboratory, La Jolla, CA, USA)[5]. Prior to docking, both protein and ligand structures underwent preprocessing with AutoDock Vina 1.1.2[6]. In the case of CYP1A2, the AutoGrid program was utilized to construct a grid box encompassing the co-crystallized ligand, ensuring comprehensive coverage of the active site of protein. The grid box had dimensions of 40 × 40 × 40 $Å^3$ and a spacing of 0.375 Å, centered on the co-crystallized ligand of CYP1A2. Default parameters were applied for other settings. Docked poses were evaluated using scoring

functions and scrutinized for protein-ligand interactions. Visualization of binding interactions was facilitated using Discovery Studio 2016 software.

18. Method validation

The bioanalytical method proposed herein was validated according to the US FDA guideline entitled 'Guidance for Industry: Bioanalytical Method Validation' (https://www.fda.gov/media/70858/download). Selectivity was evaluated by comparing the chromatograms of blank rat plasma samples, spiked blank rat plasma samples, and rat plasma samples obtained from rat pharmacokinetic studies, followed by checking for the presence of potential interferences at the acquisition windows of MHY5396 and IS. The linearity was evaluated by spiking blank plasma with increasing concentrations of MHY5396. To construct the calibration curve (n = 4), the peak area ratio of resveratrol to IS (y-axis) was plotted against the nominal concentration ratio of MHY5396 to IS (x-axis) and linear regression analysis was performed. The sensitivity was evaluated by estimating the LLOQ (based on a signal-to-noise ratio of 5). At the LLOQ, analyte peaks should be individually identified and reproduced with acceptable accuracy (80–120 %) and precision (< 20 %). The within-run and between-run precision and accuracy for each analyte were determined as previously described[7]. The accuracy and precision of the method were evaluated by comparing the measured concentrations in the QC samples (five separately prepared sets measured on one day [within-run] and five different days[between-run]) with the respective nominal concentrations, expressed as relative errors (accuracy) and respective CVs of the mean values (precision). The extraction recovery of the analytes (MHY5396 and IS) was assessed by comparing the analytical signals obtained from the extracted sample with those obtained from the post-extracted spiked sample. The matrix effect was evaluated by comparing the analytical signals obtained from the post-extracted spiked sample to those obtained from the non-extracted neat sample (diluted stock solution). Five replicates were evaluated for the LLOQ and three QC concentration levels. The stability of MHY5396 in rat plasma samples was evaluated by comparing the analytical signals obtained from the calibration standard (at LLOQ) and QC samples exposed to various handling and storage conditions with those obtained from freshly prepared plasma samples. Bench-top stability was assessed from the exposure of spiked plasma samples to room temperature for 3 h. Freeze–thaw stability was assessed from the exposure of spiked plasma samples to three complete freeze–thaw cycles (−20 °C to room temperature) on consecutive days. Long-term stability was evaluated after

storage of the spiked plasma samples at −20 °C for 30 days. Post-preparative stability (autosampler stability) was assessed from the exposure of extracted samples to 25 °C for 24 h in the autosampler.

Linearity evaluation involved plotting calibration curves for MHY5396 with nine concentration levels, demonstrating linearity within the 1–500 ng/mL range. A representative calibration curve for MHY5396 was $y = 0.2317x + 0.007$, with correlation coefficients of ≥ 0.999 across four calibration curves, indicating satisfactory linearity. Method validation parameters, including precision, accuracy, recovery, matrix effect, and stabilities (bench-top, autosampler, freeze-thaw, and long-term), are summarized in Table S4. Within-run accuracy ranged from −6.36% to 9.96%, with precision from 1.65% to 6.70%. Between-run accuracy ranged from −6.10% to 8.53%, with precision from 2.26% to 4.20%. MHY5396 recovery ranged from 90.5% to 100% with a coefficient of variation (CV) of 3.42%. Matrix effect of MHY5396 ranged from 90.8% to 104%, with a CV of 5.93%. Estimated parameter values fell within acceptable ranges, indicating precision and accuracy, along with high extraction recovery and minimal matrix effect. MHY5396 stability was assessed under various laboratory conditions encountered during bioanalytical procedures. Stability bias in measured concentrations was within ± 15% of nominal values, with remaining MHY5396 fractions ranging from 89.1% to 110%, and a CV of 9.01%. These findings suggest stability of MHY5396 under diverse handling and storage conditions.

19. In vitro protein binding and blood distribution studies

The unbound fractions of MHY5396 in plasma (fuP) liver microsomes (fuLM), and CYP1A2 isoform were measured using a rapid equilibrium dialysis device (Thermo Fisher Scientific, Inc., Waltham, MA, USA) as previously described with slight modifications[7]. Briefly, 0.2 mL of the biological matrix spiked with MHY5396 (5 µM) was placed into the "sample" chamber, and 0.4 mL of phosphate-buffered saline (PBS) was placed into the adjacent "buffer" chamber. The fraction of unbound MHY5396 was calculated as the concentration ratio between the two chambers. The blood-to-plasma concentration ratio (RB) of MHY5396 was determined as previously described[7]. Briefly, 500-µL blood spiked with MHY5396 (500 ng/mL) was incubated at 37 °C for 60 min. The blood sample was centrifuged at 2,000 × g for 5 min to obtain a plasma sample. The concentration of MHY5396 in the plasma and buffer samples was determined using the HPLC-FLD method.

20. In Situ Closed-Loop Studies in Rats

After a minimal abdominal incision under light ether anesthesia, followed by sufficient washing of the gut luminal contents, the duodenal, jejunal, ileal, and colonic closed-loops were prepared as described in our previous study[7]. MHY5396 suspension (final concentration: 0.1 mg/mL) was prepared by dispersing MHY5396 powder in the same vehicle solution used in the in vivo oral pharmacokinetic study and subsequently diluting it 5-fold with the SIF. After injecting 0.2 mL of the MHY5396 suspension into each loop using a l-mL 31-gauge syringe, the entire gut was carefully returned to its original position in the abdominal cavity. Four hours after injection, each loop was removed, transferred into a beaker containing 40 mL of methanol, and handled similarly to the gut luminal extract samples used in the in vivo pharmacokinetic study. As a control group (i.e., the "in vitro isolated loop"), the loops were prepared in the same manner as that of the "in situ loop" study. The loops were removed from the rat gut by careful cutting with scissors and transferred to a glass beaker. The MHY5396 suspension was injected into the in vitro isolated loop. After 0 and 4 h of incubation at 37°C, the remaining fraction of MHY5396 in the in vitro isolated loop was measured in the same manner as that for the gut luminal extract samples.

21. Statistical analysis for pharmacokinetic analysis

A statistical p-value was estimated using the unpaired Student's *t*-test to compare between two groups. All numerical data were presented as means rounded to three significant figures, accompanied by their respective standard deviations, excepting the median (ranges) used for T_{max}.

Supplementary tables

Supplementary Table 1. Docking score calculated based on Autodock Vina and LeDock simulation.

**FXR**		
**Program / Compound**	**Autodock Vina**	**LeDock**
Obeticholic acid	-7.4	-4.35
MHY5396	-8.1	-3.64
**PPARα**		
**Program / Compound**	**Autodock Vina**	**LeDock**
Fenofibric acid	-7.2	-3.51
MHY5396	-7.9	-3.73

Supplementary Table 2. Predicted interaction between the proteins and ligands.

	**FXR**	
	**MHY5396**	**Obeticholic acid**
**Hydrogen Bond**	THR296	THR296, TYR373
**Hydrophobic Interaction**	ALA295, MET332, ILE361, ILE366, MET369, ALA452, LEU455, MET456	LEU291, THR292, ALA295, MET332, ILE339, LEU352, ILE356, ILE361, ILE366, ALA452, LEU455, MET456, ARG459
	**PPARα**	
	**MHY5396**	**Fenofibric acid**
**Hydrogen Bond**	SER280, TYR314	CYS275
**Hydrophobic Interaction**	ILE272, PHE273, LEU344, PHE351, ILE354	ILE241, ILE272, THR279, ILE317, PHE318, LEU321, VAL332, ILE339, MET355

Supplementary Table 3. Noncompartmental plasma pharmacokinetic parameters of MHY5396 following intravenous and oral administration at 2 mg/kg in rats (n = 5).

Parameter	Intravenous	Oral
AUC_{inf} (μg·min/mL)	261 ± 47	257 ± 91
AUC_{last} (μg·min/mL)	256 ± 44	221 ± 39
$t_{1/2}$ (min)	313 ± 69	474 ± 256
T_{max} (min)		30 (15–60)
C_{max} (ng/mL)		1236 ± 273
CL (mL/min/kg)	7.88 ± 1.39	
V_{ss} (mL/kg)	1431 ± 315	
Ae_{24h} (% of dose)	1.43 ± 0.16	0.799 ± 0.290
GI_{24h} (% of dose)	0.103 ± 0.113	0.770 ± 0.642
F (%)		98.6

Supplementary Table 4. Validation parameters of HPLC analysis of MHY5396 in rat plasma (n = 5).

Nominal concentration (ng/mL)		LLOQ (1)	LQC (3)	MQC (150)	HQC (450)
Precision (%)	Within-run	5.22	6.70	1.65	1.84
	Between-run	2.38	4.20	2.26	3.09
Accuracy (%)	Within-run	100	102	103	107
	Between-run	96.5	98.5	102	106
Recovery (%)		98.1 ± 2.8	94.6 ± 1.8	94.2 ± 3.2	95.2 ± 2.2
Matrix effect (%)		97.7 ± 5.8	97.1 ± 4.2	95.0 ± 3.8	98.0 ± 5.4
Bench-top[a]		102 ± 6	103 ± 9	99.4 ± 7.0	102 ± 4
Autosampler[b]		96.0 ± 3.2	97.0 ± 3.5	100 ± 3	105 ± 5
Freeze–thaw[c]		102 ± 4	101 ± 8	107 ± 3	106 ± 2
Long-term[d]		98.7 ± 7.9	99.4 ± 4.8	98.1 ± 1.8	95.5 ± 1.3

[a] Room temperature for 3 h.

[b] 25°C for 24 h in the autosampler.

[c] Three freezing and thawing cycles.

[d] –20°C for 30 days.

Supplementary Table 5. Primer sequences for qPCR

Mouse

Gene	Forward (5'-3')	Reverse (3'-5')
Srebp1	GATCAAAGAGGAGCCAGTGC	TAGATGGTGGCTGCTGAGTG
Srebp2	GGATCCTCCCAAAGAAGGAG	TTTTTCTGATTGGCCAGCTT
Pparr	TTTTCAAGGGTGCCAGTTTC	AATCCTTGGCCCTCTGAGAT
Acca	GCCTCTTCCTGACAAACGAG	TGACTGCCGAAACATCTCTG
Fasn	TGGGTTCTAGCCAGCAGAGT	ACCACCAGAGACCGTTATGC
Scd1	TTCTTACACGACCACCACCA	GCAGGAGGGAACCAGTATGA
Tgfb	TTGCTTCAGCTCCACAGAGA	TGGTTGTAGAGGGCAAGGAC
Acta2	TTGCTGACAGGATGCAGAAG	TGATCCACATCTGCTGGAAG
Fn	CTCGGTTGTCCTTCTTGCTC	AATGGAAAAGGGGAATGGAC
Col1a1	GAGCGGAGAGTACTGGATCG	GCTTCTTTTCCTTGGGGTTC
Col1a2	GATGGCTGCTCCAAAAAGAC	CAATGTCCAGAGGTGCAATG
Vim	ATGCTTCTCTGGCACGTCTT	AGCCACGCTTTCATACTGCT
Havcr1	GTGGAAGTAAAGGGGGTGGT	TGCCCCTTTAAGTTGTACCG
Lcn2	CCAGTTCGCCATGGTATTTT	GGTGGGGACAGAGAAGATGA
Spp1	TCTGATGAGACCGTCACTGC	AGGTCCTCATCTGTGGCATC
Ccl2	TGAGGTGGTTGTGGAAAAGG	CTGGATCGGAACCAAATGAG
Ccl5	CCCTCACCATCATCCTCACT	CCTTCGAGTGACAAACACGA
Cxcl1	CCCGCGTTAGTCTGGTGTAT	AACAGCCCATAGTGGAGTGG
Emr1	ACCATCACCTATGGACCCAA	CCCAGTCATGGTCTCCAGTT
Cd163	CATGTCTCTGAGGCTGACCA	TGCACACGATCTACCCACAT
Arg1	GTGAAGAACCCACGGTCTGT	CTGGTTGTCAGGGGAGTGTT
Il1b	GCTGCTTCCAAACCTTTGAC	TTCTCCACAGCCACAATGAG
Il6	GGACCAAGACCATCCAATTC	GGCATAACGCACTAGGTTTG
Pcna	CCACATTGGAGATGCTGTTG	CAGTGGAGTGGCTTTTGTGA
Krt23	CATCTCCCTTTCCTTCACCA	CTGTGGCTGGGTTCTCTCTC
Ki67	GACAGCTTCCAAAGCTCACC	TGTGTCCTTAGCTGCCTCCT
18S rRNA	CCTGCGGCTTAATTTGACTC	AGACAAATCGCTCCACCAAC
Gapdh	AAGGTCATCCCAGAGCTGAA	CTGCTTCACCACCTTCTTGA

Rat

Gene	Forward (5’-3’)	Reverse (3’-5’)
Ccl2	ATGCAGTTAATGCCCCACTC	TTCCTTATTGGGGTCAGCAC
Cxcl1	AGACAGTGGCAGGGATTCAC	GGGGACACCCTTTAGCATCT
Il8	GAAGATAGATTGCACCGA	GATAGCCTCTCACACATTTC
Acta2	ACTGGGACGACATGGAAAAG	CATCTCCAGAGTCCAGCACA
Col1a2	CCGTGCTTCTCAGAACATCA	GAGCAGCCATCGACTAGGAC
Col3a1	AGCTGGACCAAAAGGTGATG	TCCAGTTAGCCCTGCAATTC
Acox1	TTGGAAACCACTGCCACATA	CCCGTAGCACTCTCCTTGAG
Cpt1a	ATGACGGCTATGGTGTCTCC	GTGAGGCCAAACAAGGTGAT
Cox4	ACTACCCCTTGCCTGATGTG	ACTCATTGGTGCCCTTGTTC
mtCytb	TCGAAAATCACACCCCCTAA	CGTCTCGGCAGATGTGAGTA
Dgat2	GCCGAGGTCTCTTCTCCTCT	GCACCTCAGTCTCTGGAAGG
Atp5g2	TTGACACAGCTGCCAAGTTC	GATGAGGAAGGCCACCATTA
Cox7b	CAAGTTCGAAGCATTCAGCA	AACAGGGGACAGGTTCCATT
Fasn	TCGAGACACATCGTTTGAGC	TCAAAAAGTGCATCCAGCAG
Scd1	TCCTGCTCATGTGCTTCATC	GGATGTTCTCCCGAGATTGA
Acc1	TACAACGCAGGCATCAGAAG	TGTGCTGCAGGAAGATTGAC
18s rRNA	AGTCGGCATCGTTTATGGTC	CGCGGTTCTATTTTGTTGGT
Gapdh	TGCTGGTGCTGAGTATGTCG	TTGAGAGCAATGCCAGCC

Human

Gene	Forward (5’-3’)	Reverse (3’-5’)
Acc1	ACCACCAATGCCAAAGTAGC	CTGCAGGTTCTCAATGCAAA
Fasn	CTGGCTCAGCACCTCTATCC	CAGGTTGTCCCTGTGATCCT
Dgat2	CTCTTCTTCTTCGACACCTG	TGGTCTTGTGCTTGTCGAAG
Scd1	CGACGTGGCTTTTTCTTCTC	CAAGAAAGTGGCAACGAACA
Acox1	CTGAAGGCTTTCACCTCCTG	GATGCCACACACCAACTTTC
Cpt1a	TCGTCACCTCTTCTGCCTTT	ACACACCATAGCCGTCATCA
Cox4	GGCACTGAAGGAGAAGGAGA	GGGCCGTACACATAGTGCTT
Ndufs1	CAGGGAAGGTGTGATGGAGT	CAAACCTGATGCAGCGAGTA
Atp5	GCCTGCTCCAAGTTTGTCTC	TGTGTCGATGTCCCTTGAAA
mtCytb	AGGCGTCCTTGCCCTATTAC	ACTGGTTGTCCTCCGATTCA
Col1a2	CTGCAAGAACAGCATTGCAT	GGCGTGATGGCTTATTTGTT

Fn	ACCAACCTACGGATGACTCG	GCTCATCATCTGGCCATTTT
Vim	GAGAACTTTGCCGTTGAAGC	TCCAGCAGCTTCCTGTAGGT
18S rRNA	ATGGCCGTTCTTAGTTGGTG	CGCTGAGCCAGTCAGTGTAG

Supplementary Table 6. Information of primary antibodies used in Western blotting

Antibody	Company	Catalog number
FXR	Santa Cruz Biotechnology	sc-25309
PPARα	Novusbio	NB600-636
Lamin A/C	Santa Cruz Biotechnology	sc-376248
αSMA	Santa Cruz Biotechnology	sc-32251
GAPDH	Santa Cruz Biotechnology	sc-365062
Collagen1α2	Santa Cruz Biotechnology	sc-393573
α-Tubulin	Santa Cruz Biotechnology	sc-5286
Vimentin	Cell Signaling Technology	#5741S
p65	Santa Cruz Biotechnology	sc-514451
p-p65	Santa Cruz Biotechnology	sc-136548
Smad2/3	Santa Cruz Biotechnology	sc-133098
pSmad2	Santa Cruz Biotechnology	sc-135644
pSmad3	Abcam	ab52903
Acox1	Abcam	ab184032
Cpt1α	Proteintech	15184
OXPHOS	Abcam	ab110413

Supplementary figures

Supplementary Figure 1. Structures of benzoxazole derivatives tested for dual agonist. Structure of MHYs, a dual agonist of the farnesoid X receptor (FXR) and peroxisome proliferator-activated receptor (PPAR) alpha, based on the benzoxazole scaffold.

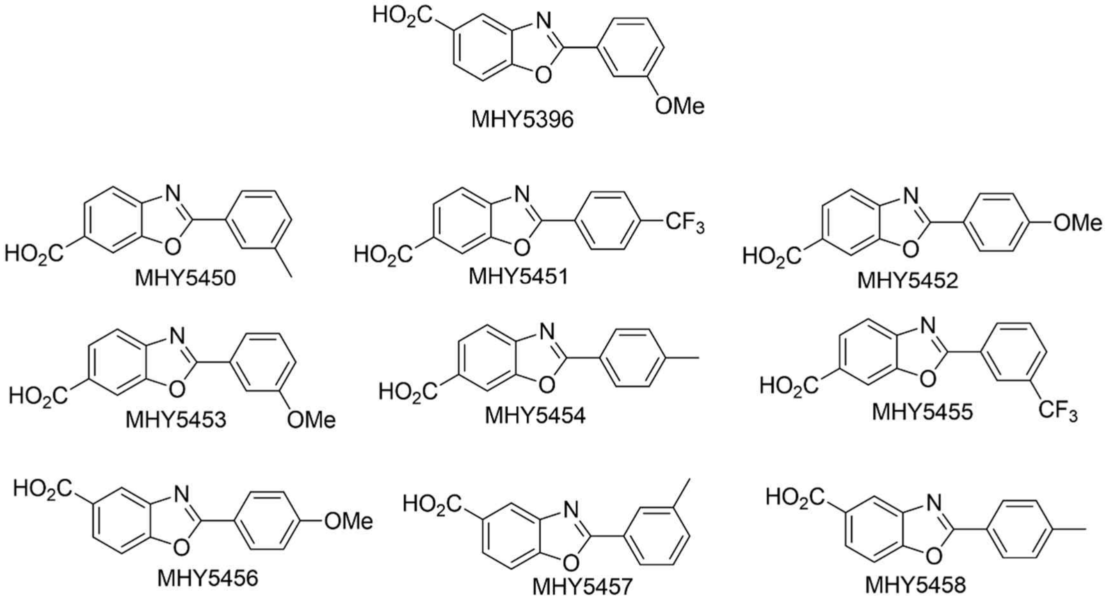

Supplementary Figure 2. Thermal shift assay results of FXR and PPARα ligand-binding domains (LBDs) in the presence of ligands. (A) Thermal stability of FXR LBD in the absence or presence of OCA (20 μM) or MHY5396 (20 μM). (B) Thermal stability of PPARα LBD in the absence or presence of fenofibric acid (Feno, 20 μM) or MHY5396 (20 μM).

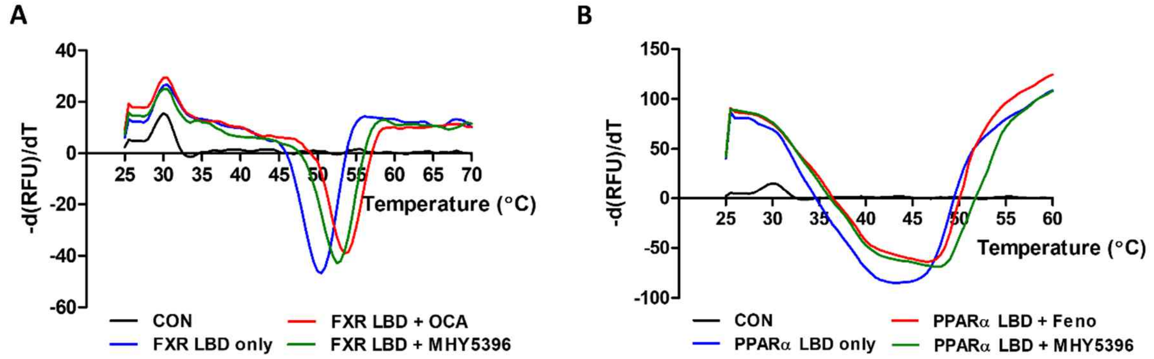

Supplementary Figure 3. Pharmacophore analysis performed based on interactions between the proteins and ligands. Pharmacophore analysis of (a) obeticholic acid and (b) MHY5396 against FXR, and (c) fenofibrate and (d) MHY5396 against PPARα. Green arrows indicate hydrogen bond (H-bond) donors, red arrows indicate H-bond acceptors, and yellow regions indicate hydrophobic interactions or van der Waals forces. The involved amino acid residues are alanine (ALA), arginine (ARG), isoleucine (ILE), leucine (LEU), methionine (MET), phenylalanine (PHE), serine (SER), threonine (THR), tyrosine (TYR), and valine (VAL).

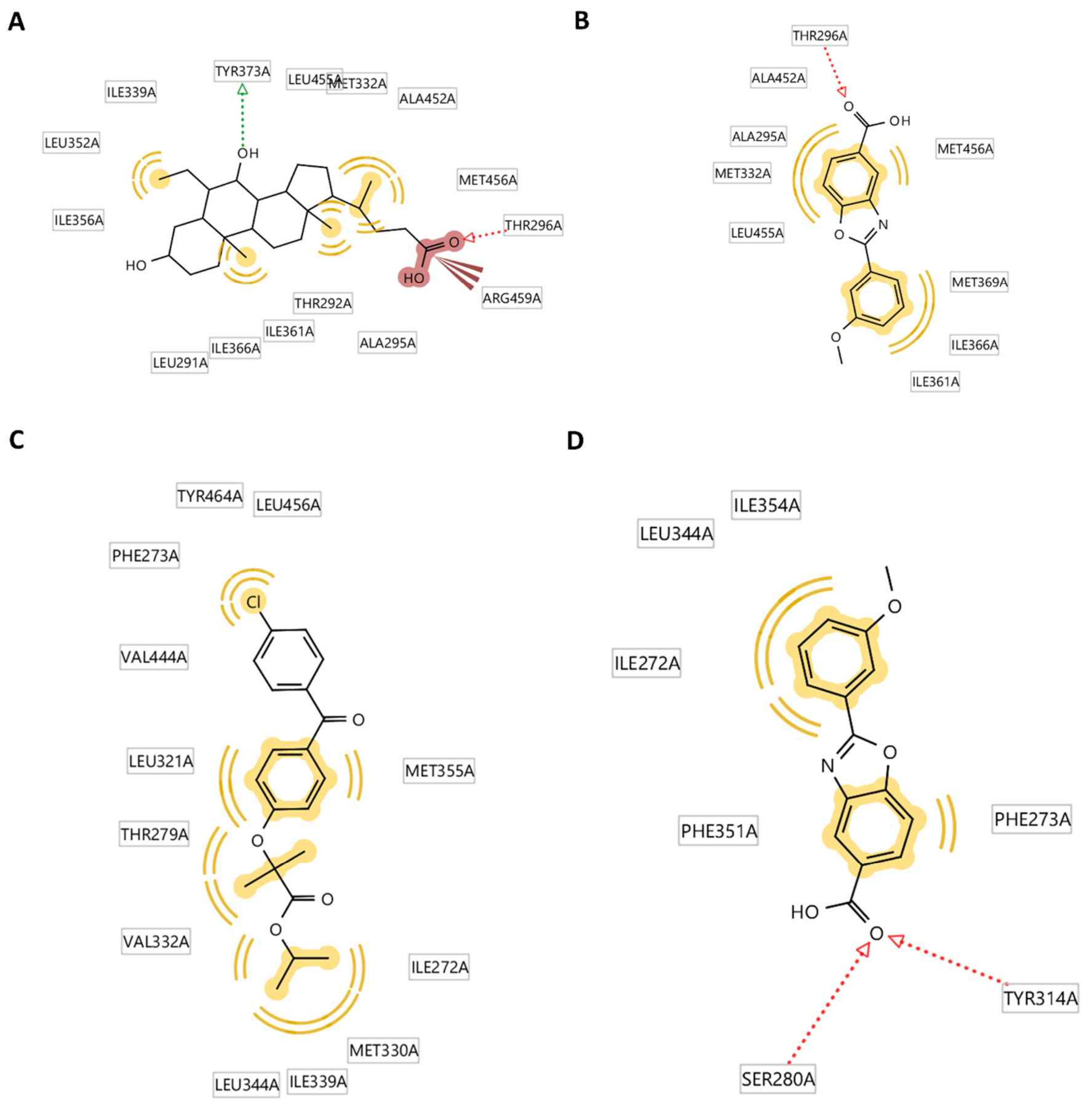

Supplementary Figure 4. Docking simulation results of MHY5396 on PPARα and FXR using DiffDock-L. 3D docking structures of the compound MHY5396 with the nuclear receptors PPARα (A) (Peroxisome Proliferator-Activated Receptor Alpha) and FXR (B) (Farnesoid X Receptor). Polar contacts with MHY5396 highlighted.

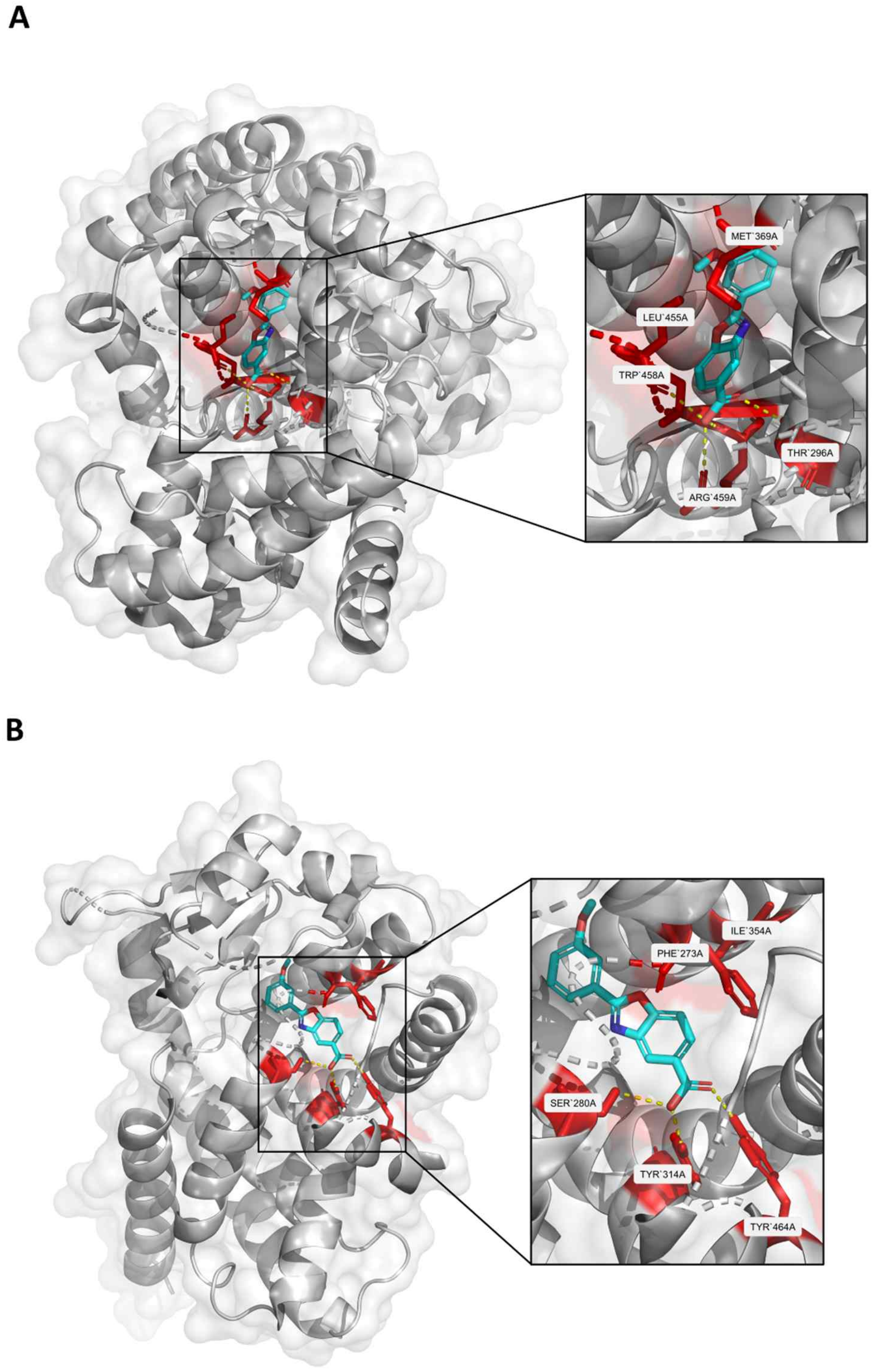

Supplementary Figure 5. Effect of MHY5396 on lipid metabolism in AC2F liver hepatocytes. (A) Schematic diagram of the experiment to confirm genetic changes caused by single treatment with MHY5396 and alterations in oleic acid (OA)-induced lipid metabolism by MHY5396 treatment in AC2F cells. (B) Relative mRNA levels of lipid metabolism-related genes (*Acc1, Fasn, Dgat2*, and *Scd1*) in MHY5396-single treated AC2F cells. The results are quantified as a ratio to 18S rRNA. *P < 0.05 compared to the control group. (C) Relative mRNA levels of β-oxidation-related genes (*Acox1* and *Cpt1a*) in MHY5396-single treated AC2F cells. The results are quantified as a ratio to 18S rRNA. *P < 0.05 compared to the control group. (D) Triglyceride (TG) levels in AC2F cells treated with OA, with or without MHY5396. ###P < 0.001 compared to the control group. ***P < 0.001 compared to the OA-treated group. (E) Representative images of Oil red O (ORO) staining of AC2F cells.

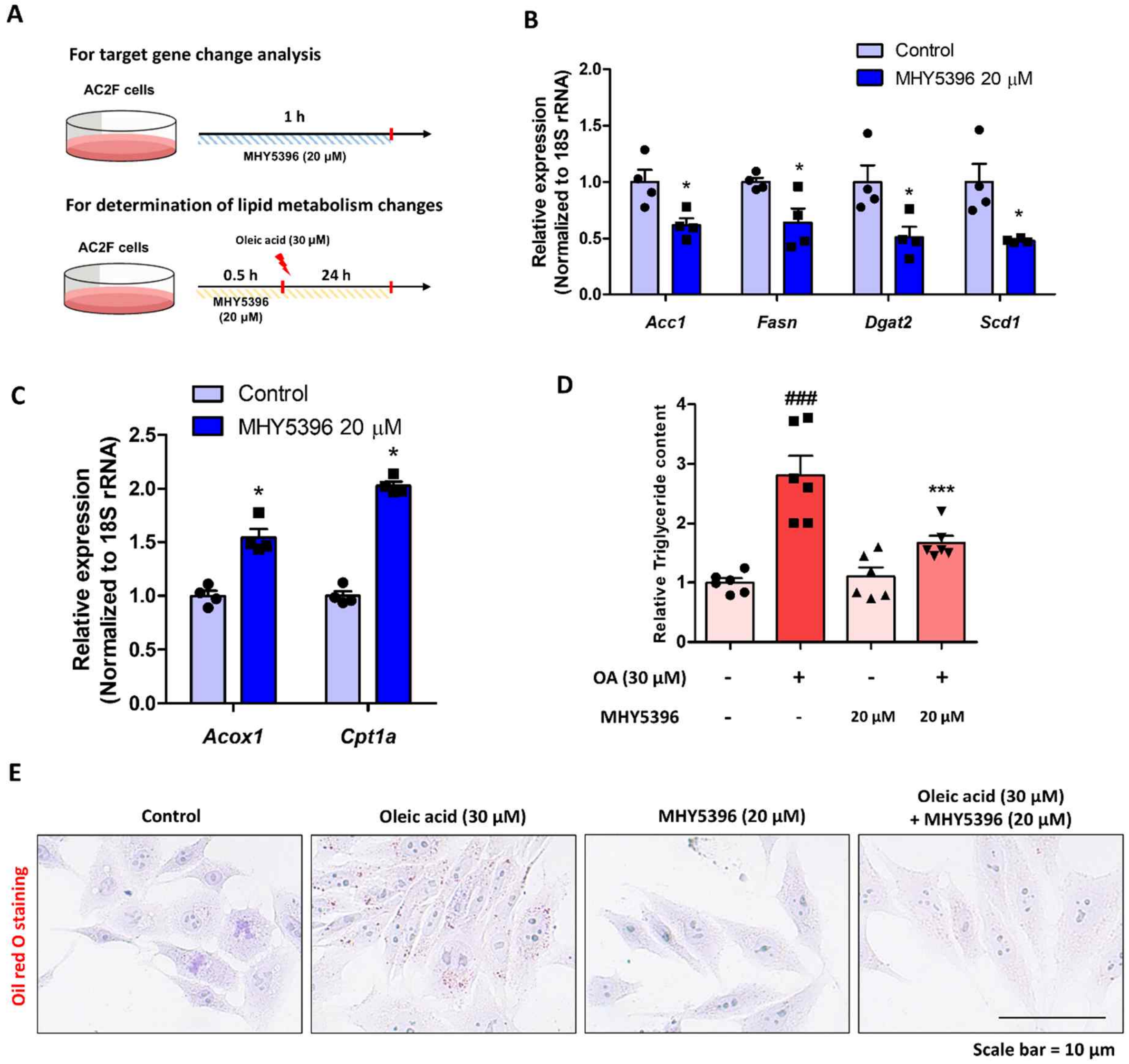

Supplementary Figure 6. Effect of obeticholic acid and fenofibric acid on TGFβ-induced fibrosis response in LX2 cells and oleic acid-induced lipid accumulation in hepatocytes. (A) Schematic diagram of the experiment validating the anti-fibrotic effect of obeticholic acid (OCA) and fenofibric acid in TGFβ-treated LX2 stellate cells. (B) Relative mRNA levels of fibrosis-related genes (*Acta2, Col1a2, Col3a1 Fn*, and *Vim*) in TGFβ-treated LX2 cells with or without OCA treatment. The results are quantified as ratios of *Gapdh*. *p<0.05 compared to the control group. ***p<0.001 compared to the control group. #p<0.05 compared to the TGFβ-treated group. ##p<0.005 compared to the TGFβ-treated group. ##p<0.001 compared to the TGFβ-treated group. (C) Relative mRNA levels of fibrosis-related genes (*Acta2, Col1a2, Col3a1, Fn*, and *Vim*) in TGFβ-treated LX2 cells with or without fenofibric acid treatment. The results are quantified as ratios of *Gapdh*. ***p<0.001 compared to the control group. #p<0.05 compared to the TGFβ-treated group. ##p<0.005 compared to the TGFβ-treated group. ##P<0.005 compared to the TGFβ-treated group. (D) Schematic diagram of the experiment assessing the effects of OCA and fenofibric acid on oleic acid-induced lipid accumulation in AC2F cells. (E) Relative triglyceride levels in OA with or without OCA treatment in AC2F cells. ###p<0.001 compared to the control group. *p<0.05 compared to the OA-treated group. (F) Relative triglyceride levels in OA with or without fenofibric acid treatment in AC2F cells. ###p<0.001 compared to the control group. ***p<0.001 compared to the OA-treated group.

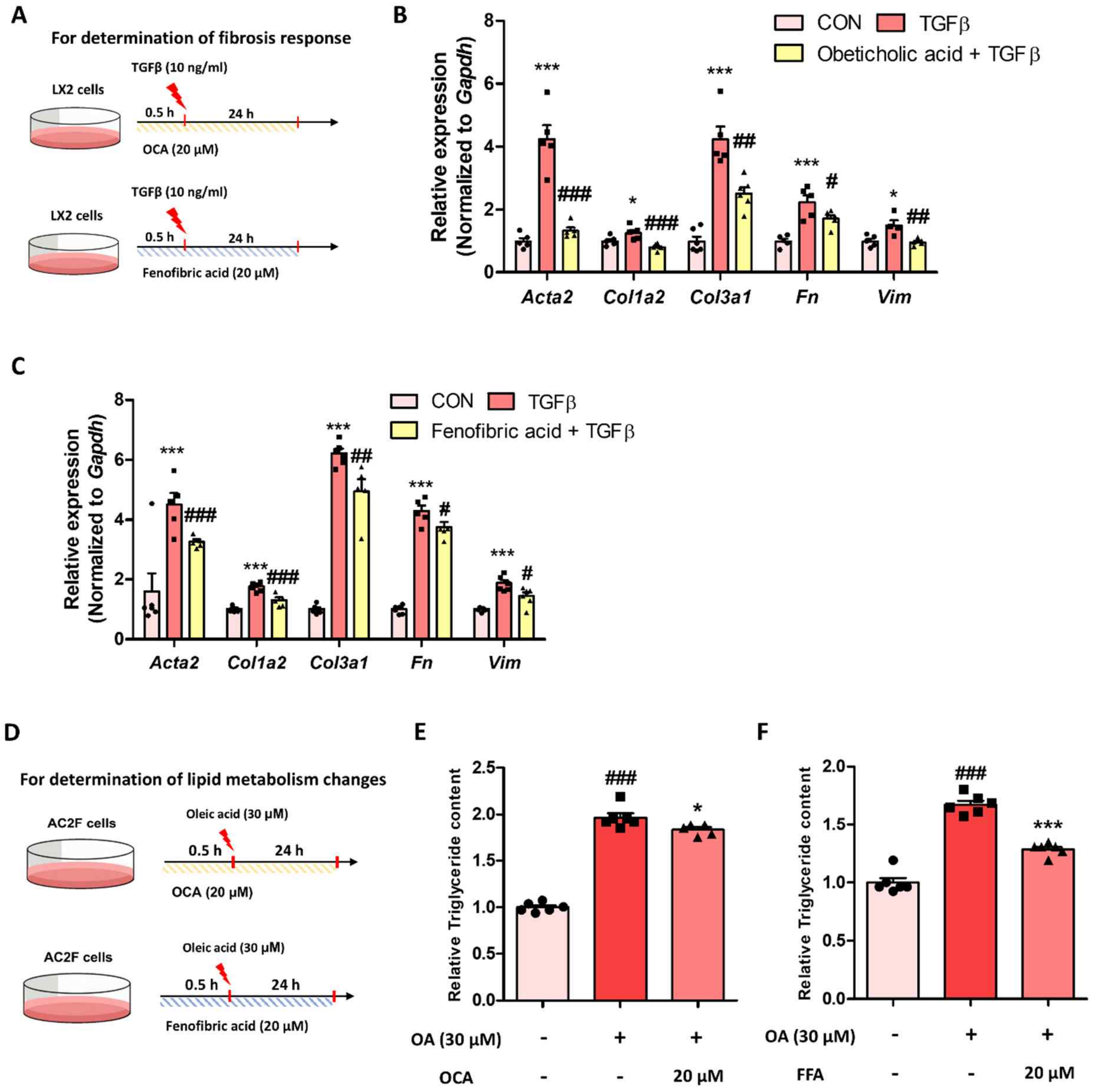
A
For determination of fibrosis response
TGFβ (10 ng/ml)
LX2 cells
0.5 h
24 h
OCA (20 μM)
TGFβ (10 ng/ml)
LX2 cells
0.5 h
24 h
Fenofibric acid (20 μM)
B
CON
TGFβ
Obeticholic acid + TGFβ
Relative expression
(Normalized to Gapdh)
***
###
*
###
***
##
***
#
*
##
Acta2
Col1a2
Col3a1
Fn
Vim
C
CON
TGFβ
Fenofibric acid + TGFβ
Relative expression
(Normalized to Gapdh)
***
###
***
###
***
##
***
#
***
#
Acta2
Col1a2
Col3a1
Fn
Vim
D
For determination of lipid metabolism changes
AC2F cells
Oleic acid (30 μM)
0.5 h
24 h
OCA (20 μM)
AC2F cells
Oleic acid (30 μM)
0.5 h
24 h
Fenofibric acid (20 μM)
E
Relative Triglyceride content
###
*
OA (30 μM)
OCA
20 μM
F
Relative Triglyceride content
###
***
OA (30 μM)
FFA
20 μM

Supplementary Figure 7. Effect of a single oral administration of MHY5396 in mice. (A) MHY5396 (5 mg/kg) was administered orally to mice, which were sacrificed 6 hours after treatment. (B) Relative mRNA expression levels of hepatic β-oxidation–related genes (*Ppara, Acox1,* and *Cpt1a*) in the presence or absence of MHY5396 treatment. Gene expression levels were normalized to 18S rRNA. *p < 0.05 compared to the non-treated group. (C) Relative mRNA expression levels of lipid synthesis–related genes (*Acca, Scd1, Lpin1*, and *Chrebp*) with or without MHY5396 treatment. Gene expression levels were normalized to 18S rRNA. *p < 0.05 compared to the non-treated group.

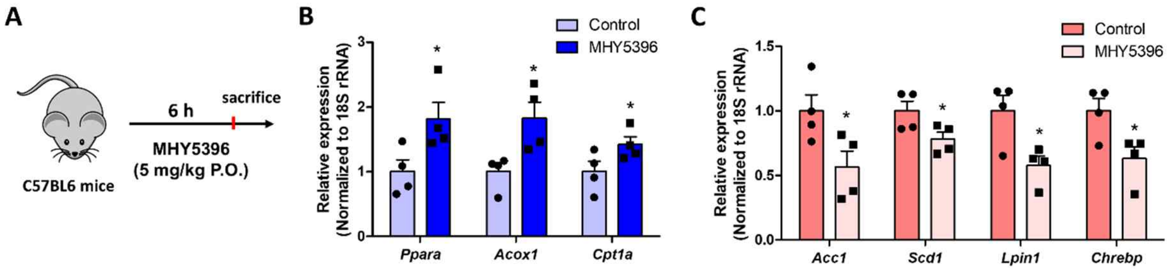

Supplementary Figure 8. Effect of MHY5396 on MCD-induced body weight changes, histological and fibrotic changes in liver. (A) Final mouse body weight on the last day of the experiment. (B) Serum ALT levels in MCD diet mice with or without MHY5396 treatment. ***p<0.001 compared to the control mouse group. #p<0.05 compared to the MCD diet mouse group. (C) Representative hematoxylin & eosin (H&E) staining of mouse liver tissue sections. (D) Relative mRNA levels of hepatic lipid metabolism-related genes (*Srebp1, Srebp2, Pparr. Acca. Fasn*, and *Scd1*) with or without MHY5396 treatment. The results are quantified as ratios of 18S rRNA. *p<0.05 compared to the MCD diet mouse group. (E) Relative mRNA levels of hepatic fibrosis-related genes (*Tgfb1, Acta2, Fn*, and *Col1a1*) with or without MHY5396 treatment. The results are quantified as ratios 18S rRNA. *p<0.05 compared to the MCD diet mouse group. (F) Representative in situ hybridization (ISH) images detected with a Vimentin (*Vim*, red) probe in methionine choline deficient (MCD) diet-induced mouse liver with or without MHY5396 treatment (Scale bar = 100 μm).

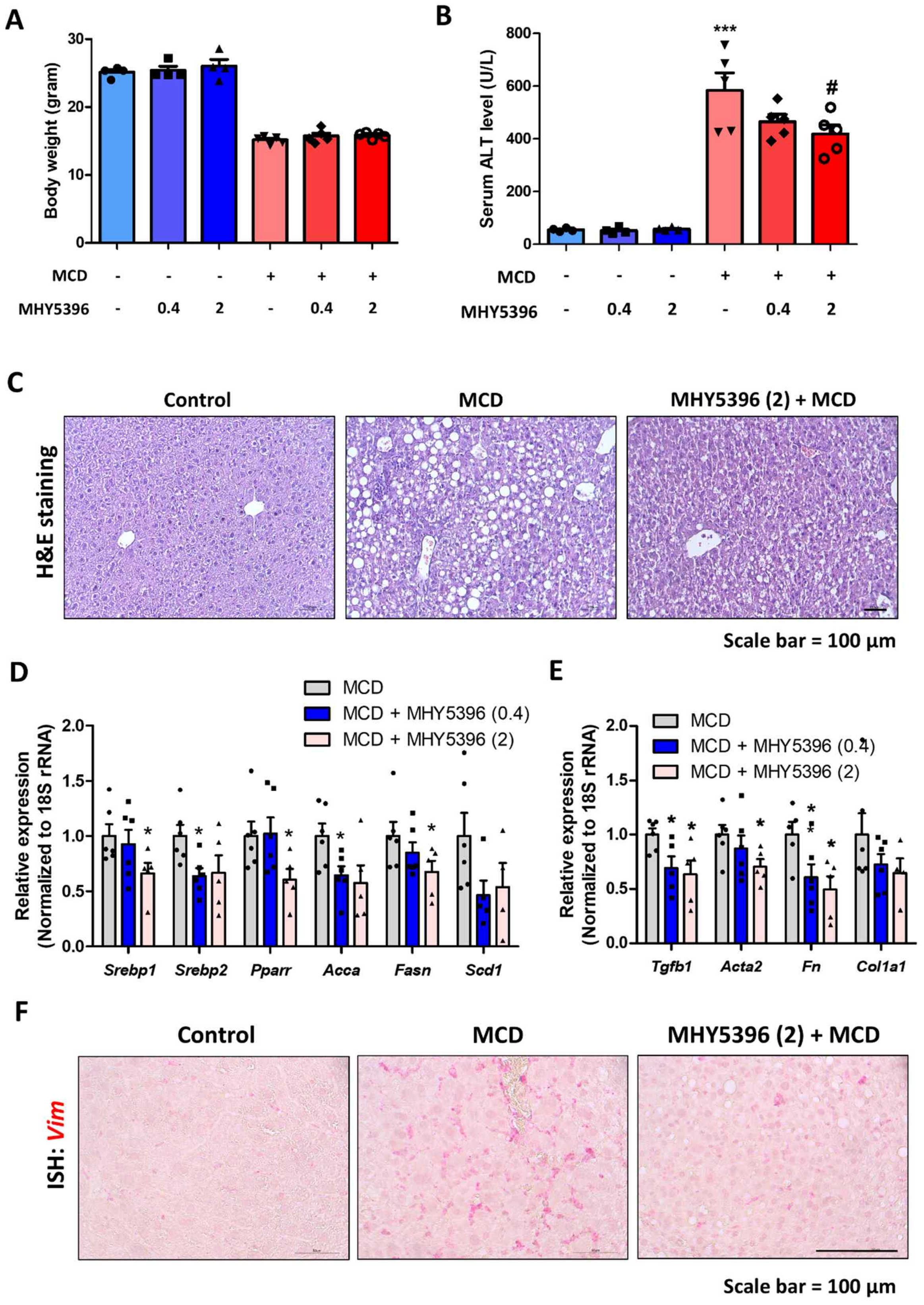
A
Body weight (gram)
30
20
10
0
MCD - - - + + +
MHY5396 - 0.4 2 - 0.4 2
B
Serum ALT level (U/L)
800
600
400
200
0
***
#
MCD - - - + + +
MHY5396 - 0.4 2 - 0.4 2
C
Control
MCD
MHY5396 (2) + MCD
H&E staining
Scale bar = 100 μm
D
MCD
MCD + MHY5396 (0.4)
MCD + MHY5396 (2)
Relative expression
(Normalized to 18S rRNA)
2.0
1.5
1.0
0.5
0.0
Srebp1
Srebp2
Pparr
Acca
Fasn
Scd1
E
MCD
MCD + MHY5396 (0.4)
MCD + MHY5396 (2)
Relative expression
(Normalized to 18S rRNA)
2.0
1.5
1.0
0.5
0.0
Tgfb1
Acta2
Fn
Col1a1
F
Control
MCD
MHY5396 (2) + MCD
ISH: Vim
Scale bar = 100 μm

Supplementary Figure 9. Effect of MHY5396 on Pcna, Krt23, and Ki67 gene expression in liver. (A) Relative mRNA levels of Pcna following MHY5396 administration. The results are quantified as a ratio to 18S rRNA. (B) Relative mRNA levels of Krt23 following MHY5396 administration. The results are quantified as a ratio to 18S rRNA. (C) Relative mRNA levels of Ki67 following MHY5396 administration. The results are quantified as a ratio to 18S rRNA.

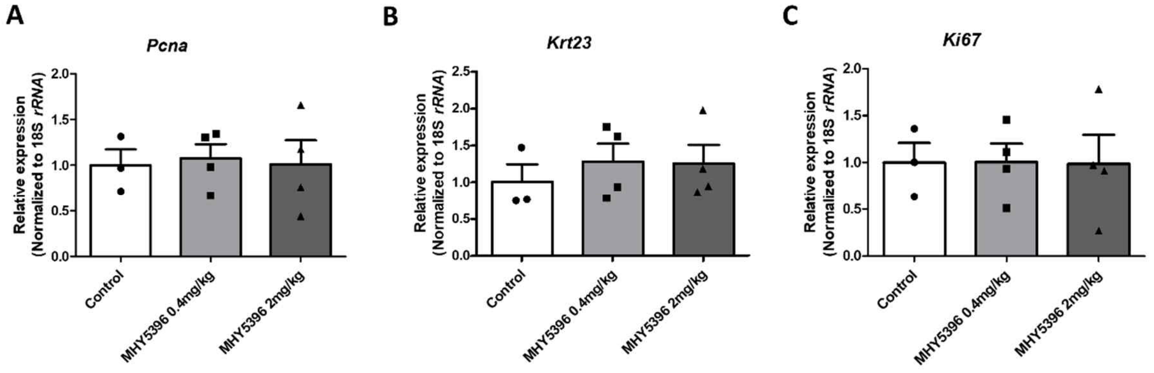

Supplementary Figure 10. Effect of MHY5396 on high-fat diet (HFD)-induced body weight changes and fatty liver development. (A) Experimental scheme illustrating HFD-induced fatty liver and the effect of MHY5396 (n = 6). (B) Final mouse body weight at the end of the experiment. **p < 0.005 compared to the control group. (C) Hepatic triglyceride (TG) levels in the livers of HFD-fed mice with or without MHY5396 treatment. ***p < 0.001 compared to the control group; #p < 0.05, ##p < 0.005 compared to the HFD group. (D) Representative Oil Red O (ORO) staining of mouse liver sections. (E) Relative mRNA expression levels of hepatic lipid metabolism-related genes (*Acca, Fasn, Scd1*, and *Ppara*) with or without MHY5396 treatment. Results are normalized to 18S rRNA. #p < 0.05 compared to the HFD group.

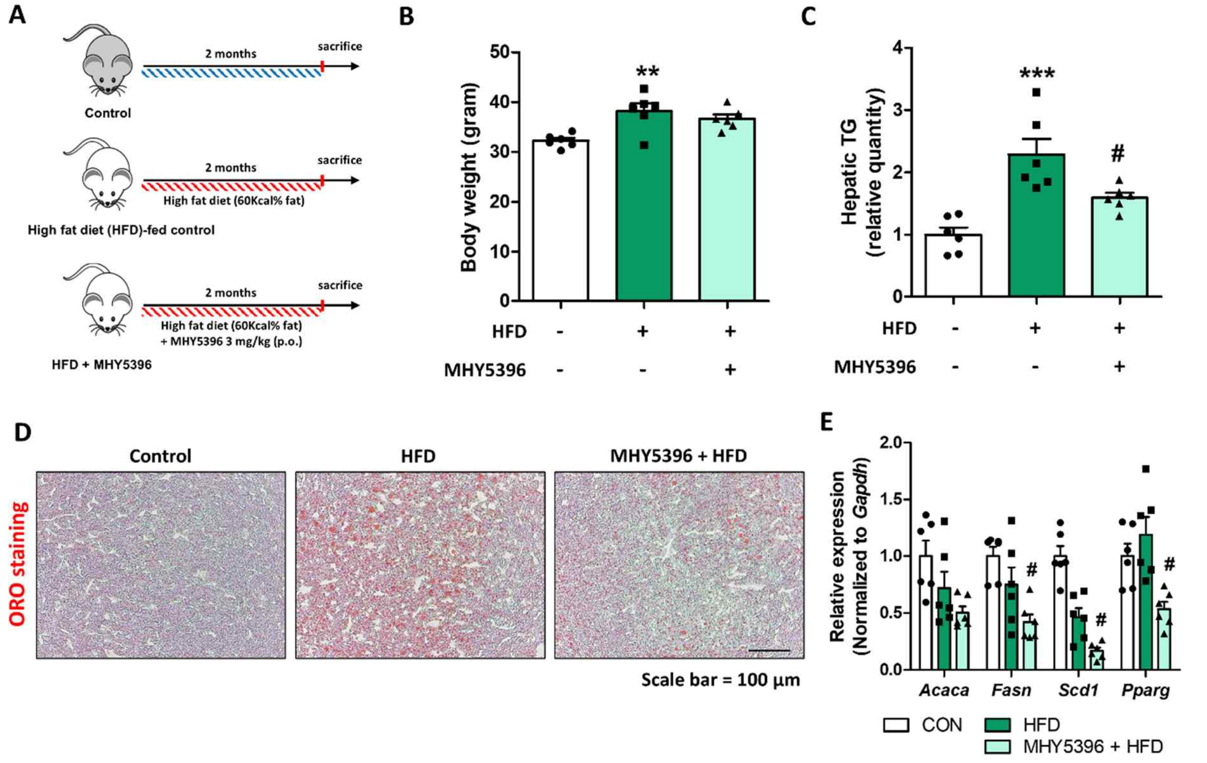

Supplementary Figure 11. MHY5396 alleviates thioacetamide (TAA)-induced liver fibrosis in mice when administered post-treatment. (A) Experimental scheme to assess the therapeutic effect of MHY5396 in TAA-induced established liver fibrosis model (n=7-10). (B) Representative H&E staining of mouse liver tissue sections. (C) Serum AST levels in TAA-treated mice with or without MHY5396 treatment. **p<0.005 compared to the control mouse group. #p<0.05 compared to the TAA-treated group. (D) Relative mRNA levels of hepatic fibrosis-related genes (*Acta2, Col1a2, Col3a1, Tgfb,* and *Vim*) with or without MHY5396 treatment. The results are quantified as ratios of *Gapdh*. **p<0.005, ***p<0.001 compared to the control mouse group. #P<0.05 compared to the TAA-injected mouse group. (E) Col1a1, and Vimentin protein levels were determined in TAA-injected mouse livers with or without MHY5396 treatment. α-tubulin was used as the loading control. Relative protein expressions were quantified using densitometry. ***p<0.001 compared to the control group. #p<0.05, ###p<0.001 compared to the TAA-treated group. (F) Representative SR staining of mouse liver tissue sections. Positive areas for SR staining were calculated to determine the extent of hepatic fibrosis in each experimental group. ***p<0.001 compared to the control mouse group. ###p<0.001 compared to the TAA-injected mouse group.

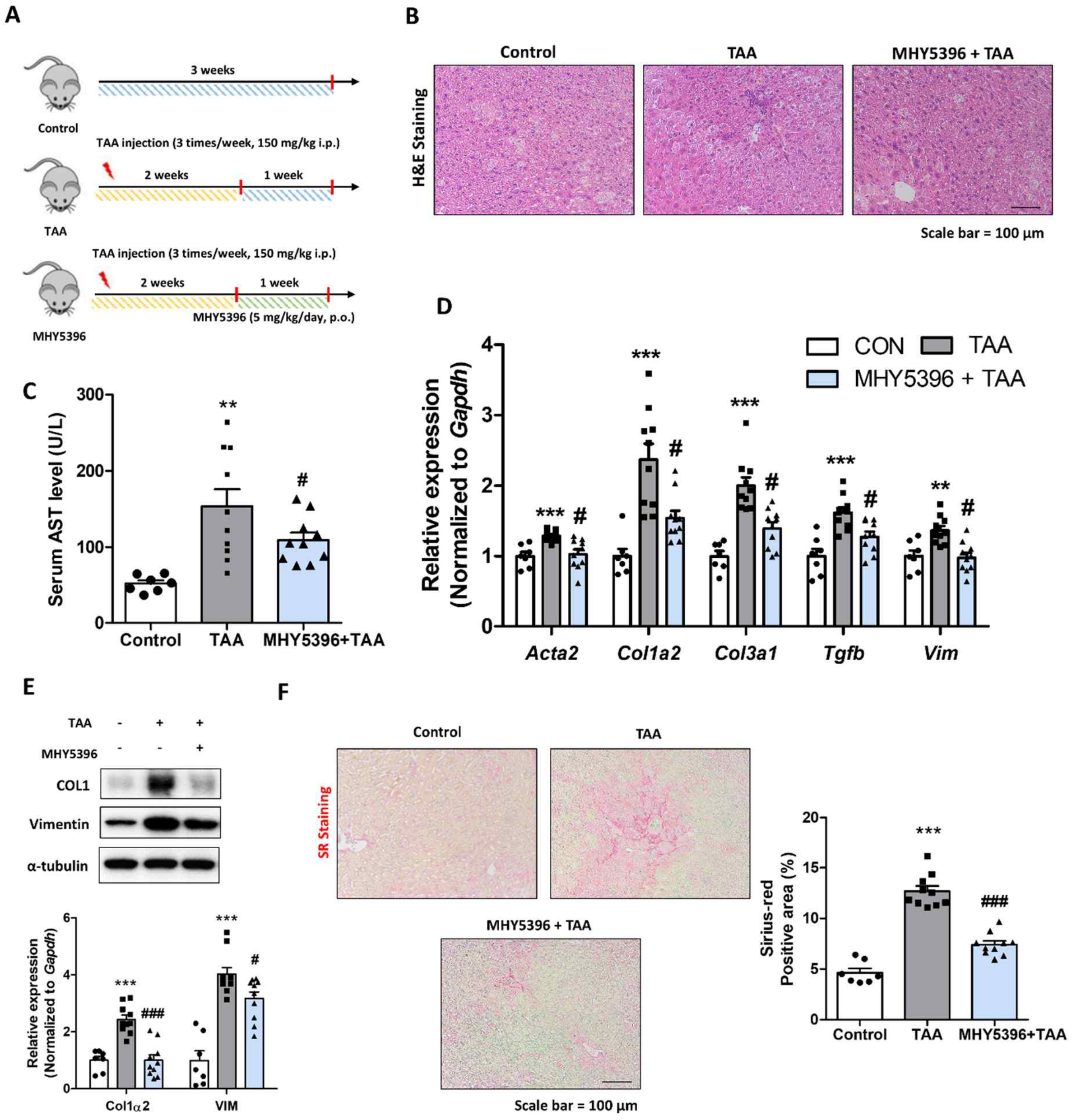
A
3 weeks
Control
TAA injection (3 times/week, 150 mg/kg i.p.)
2 weeks
1 week
TAA
TAA injection (3 times/week, 150 mg/kg i.p.)
2 weeks
1 week
MHY5396 (5 mg/kg/day, p.o.)
MHY5396
B
Control
TAA
MHY5396 + TAA
H&E Staining
Scale bar = 100 µm
C
Serum AST level (U/L)
Control
TAA
MHY5396+TAA
D
Relative expression (Normalized to Gapdh)
CON
TAA
MHY5396 + TAA
Acta2
Col1a2
Col3a1
Tgfb
Vim
E
TAA
MHY5396
COL1
Vimentin
α-tubulin
Relative expression (Normalized to Gapdh)
Col1α2
VIM
F
Control
TAA
MHY5396 + TAA
SR Staining
Scale bar = 100 µm
Sirius-red Positive area (%)
Control
TAA
MHY5396+TAA

Supplementary Figure 12. Effect of MHY5396 on cell viability of NRK52E cells and TGFβ-induced Smad signaling changes in NRK49F cells. (A) The cytotoxicity of MHY5396 was evaluated in a dose-dependent manner in NRK52E cells. Results were expressed as percentages. (B) Protein levels of Smad signaling proteins (such as Smad2/3, pSmad2, and pSmad3) were determined in NRK49F cells treated with TGFβ, with or without MHY5396 treatment. GAPDH was used as a loading control. Relative protein expressions were quantified using densitometry. **p<0.005, ***p<0.001 compared to the control group.

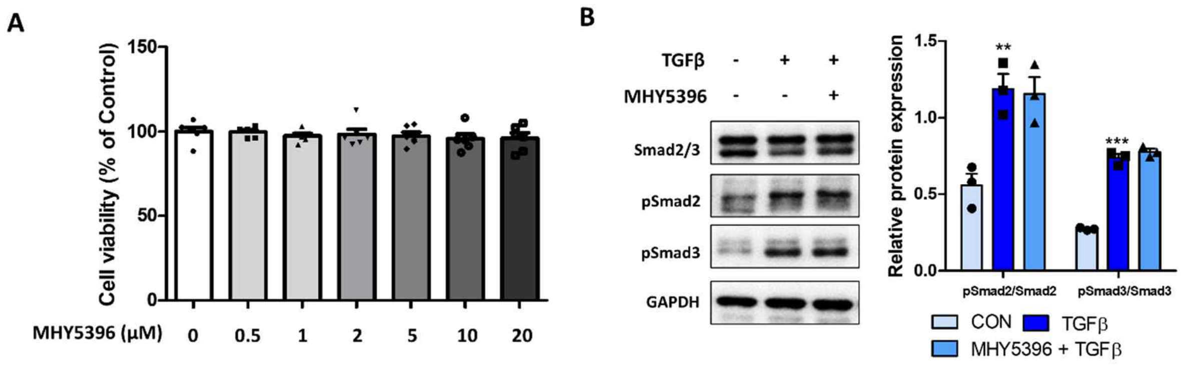

Supplementary Figure 13. MHY5396 modulates lipid metabolism in NRK52E renal epithelial cells. (A) Schematic diagram of the experiment to confirm genetic changes caused by single treatment with MHY5396 and alterations in oleic acid (OA)-induced lipid metabolism by MHY5396 treatment in NRK52E cells. (B) Relative mRNA levels of lipid metabolism-related genes (*Acc1, Fasn,* and *Scd1*) in MHY5396-single treated NRK52E cells. The results are quantified as a ratio to 18S rRNA. *p < 0.05 compared to the control group. (C) Relative mRNA levels of β-oxidation-related genes (*Acox1* and *Cpt1a*) in MHY5396-single treated NRK52E cells. The results are quantified as a ratio to 18S rRNA. *p < 0.05 compared to the control group. (D) Relative mRNA levels of mitochondria-related genes (such as *Cox4, Cox7b, Atp5g2*, and *mtCytb*) in MHY5396-single treated NRK52E cells. The results are quantified as a ratio to 18S rRNA. * p < 0.05 compared to the control group. (E) Triglyceride (TG) levels in NRK52E cells treated with OA, with or without MHY5396. ###p < 0.001 compared to the control group. **p < 0.005 compared to the OA-treated group. (F) Representative images of Oil red O (ORO) staining of NRK52E cells.

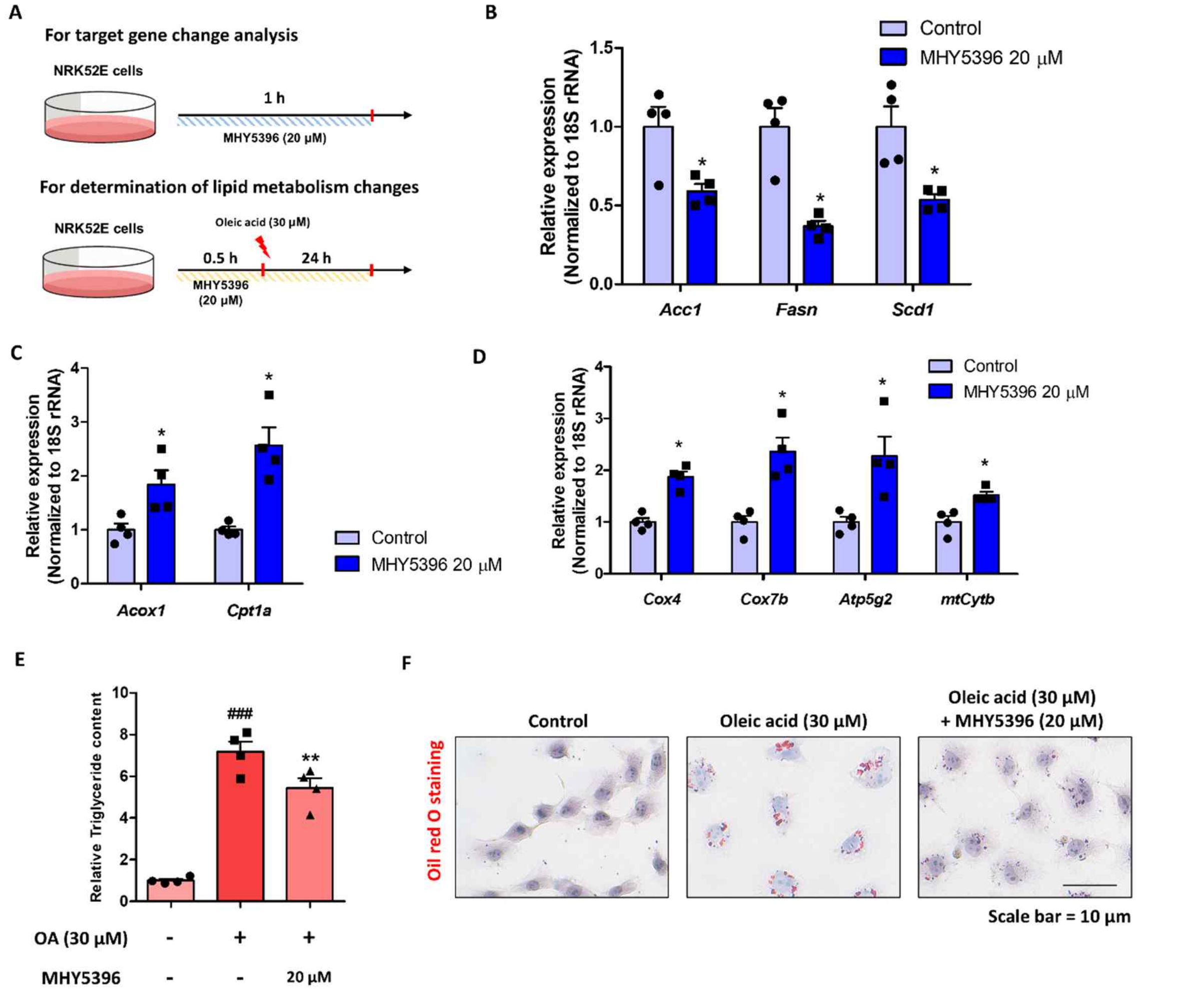

Supplementary Figure 14. MHY5396 decreases lipid accumulation induced by FA-treatment and increases OXPHOS, β-oxidation-related proteins in kidney. (A) Triglyceride (TG) levels in folic acid (FA)-induced mouse kidneys with or without MHY5396 treatment. The results are quantified as a ratio to kidney weight. ###p < 0.001 compared to the control group. **p < 0.005, ***p < 0.001 compared to the FA-treated group. (B) Protein levels of β-oxidation-related proteins (OXPHOS, *Acox1,* and *Cpt1α*) were determined in FA-induced mouse kidneys with or without MHY5396 treatment. α-tubulin was used as a loading control. Relative protein expressions were quantified using densitometry. *p<0.05, **p<0.005 compared to the control group. #p<0.05 compared to the FA-treated group.

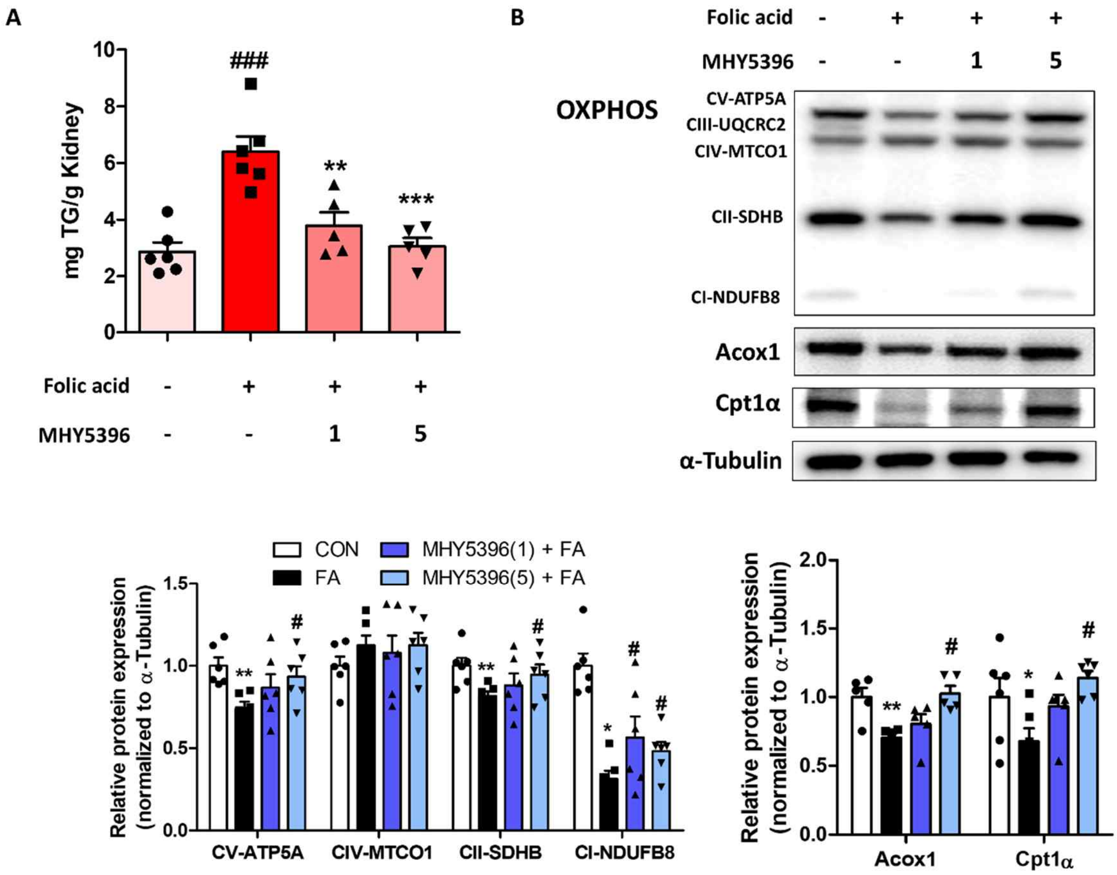

Supplementary Figure 15. MHY5396 mitigates established renal fibrosis induced by an adenine diet (AD) in mice. (A) Experimental scheme to assess the therapeutic effect of MHY5396 in AD-induced established renal fibrosis model (n=4-5). (B) Serum creatinine levels in AD-fed mice with or without MHY5396 treatment. **p<0.005 compared to the control mouse group. #p<0.05 compared to the AD-treated mouse group. (C) Relative mRNA levels of kidney damage-related genes (*Havcr1, Spp1*) in AD-fed mouse kidneys with or without MHY5396 treatment. ***p<0.001 compared to the control mouse group. #p<0.05, compared to the AD-fed mouse group. (D) Representative H&E staining of mouse kidney tissue sections. (E) Relative mRNA levels of kidney inflammation-related genes (*Il1b, Il6, and Ccl2*) in AD-fed mouse kidneys with or without MHY5396 treatment. ***p<0.001 compared to the control mouse group. #p<0.05 compared to the AD-fed mouse group. (F) Protein levels of p65 and p-p65 in AD-fed mouse kidneys with or without MHY5396 treatment. α-tubulin was used as the loading control. Relative protein expressions were quantified using densitometry. *p<0.05 compared to the control group. #p<0.05 compared to the AD-fed group. (G) Relative mRNA levels of macrophage marker genes (*Emr1, Arg1*) in AD-fed mouse kidneys with or without MHY5396 treatment. ***p<0.001 compared to the control mouse group. #p<0.05 compared to the AD-fed mouse group. (H) Relative mRNA levels of fibrosis-related genes (*Col1a2, Tgfb*) in AD-fed mouse kidneys with or without MHY5396 treatment. ***p<0.001 compared to the control mouse group. #p<0.05 compared to the AD-fed mouse group. (I) Protein levels of COL1 and VIM in AD-fed mouse kidneys with or without MHY5396 treatment. α-tubulin was used as the loading control. Relative protein expressions were quantified using densitometry. *p<0.05, ***p<0.001 compared to the control group. #p<0.05 compared to the AD-fed group. (J) Representative images of SR staining of mouse kidney tissue sections.

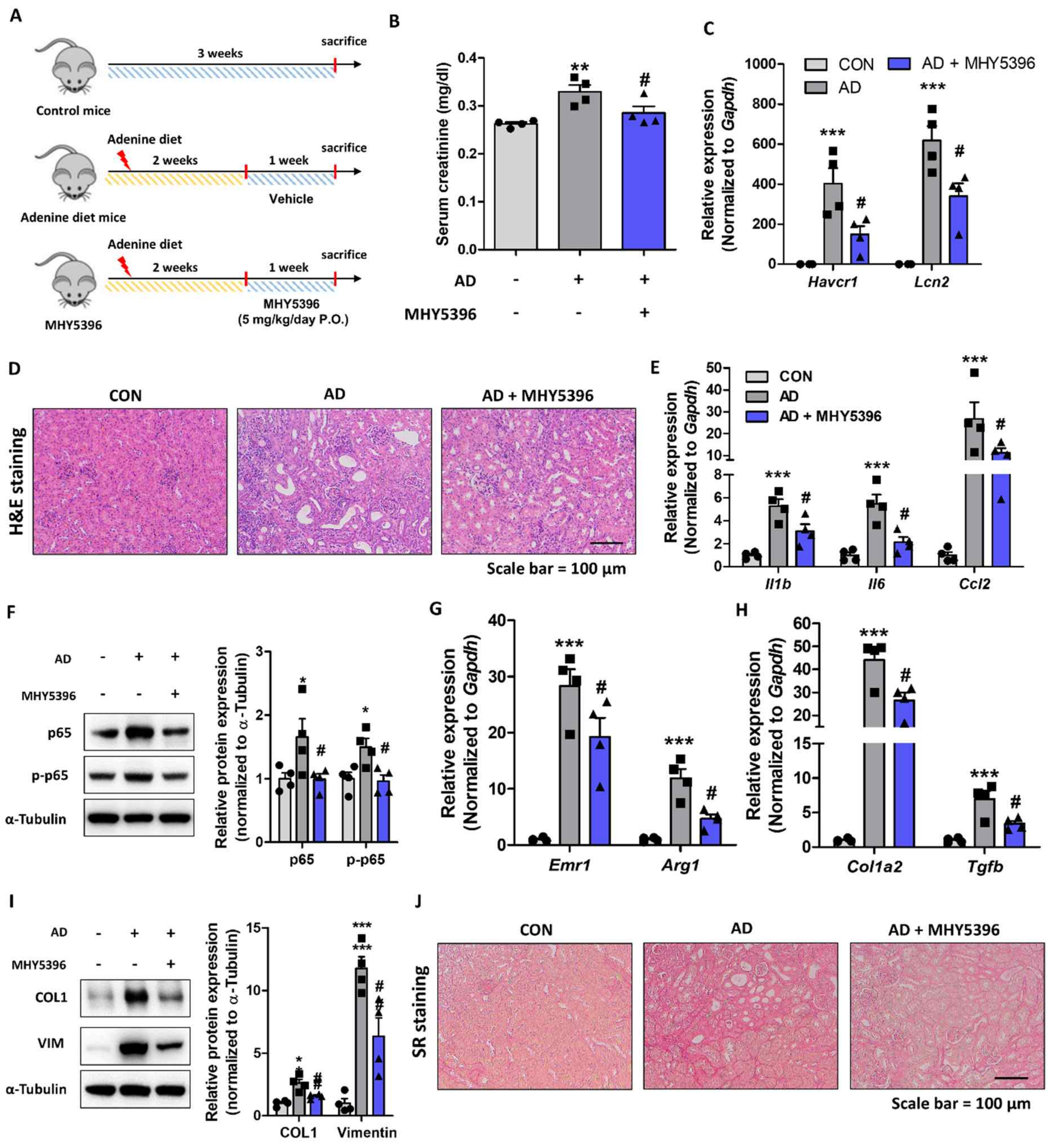

A
3 weeks
sacrifice
Control mice
Adenine diet
2 weeks
1 week
sacrifice
Vehicle
Adenine diet mice
Adenine diet
2 weeks
1 week
sacrifice
MHY5396
(5 mg/kg/day P.O.)
MHY5396
B
Serum creatinine (mg/dl)
AD
MHY5396
C
CON
AD
AD + MHY5396
Relative expression (Normalized to Gapdh)
Havcr1
Lcn2
D
CON
AD
AD + MHY5396
H&E staining
Scale bar = 100 μm
E
CON
AD
AD + MHY5396
Relative expression (Normalized to Gapdh)
Il1b
Il6
Ccl2
F
AD
MHY5396
p65
p-p65
α-Tubulin
Relative protein expression (normalized to α-Tubulin)
G
Relative expression (Normalized to Gapdh)
Emr1
Arg1
H
Relative expression (Normalized to Gapdh)
Col1a2
Tgfb
I
AD
MHY5396
COL1
VIM
α-Tubulin
Relative protein expression (normalized to α-Tubulin)
COL1
Vimentin
J
CON
AD
AD + MHY5396
SR staining
Scale bar = 100 μm

Supplementary Figure 16. Benzoxazole derivatives as potent FXR and PPAR alpha dual agonists with anti-fibrosis and metabolic regulatory effect.

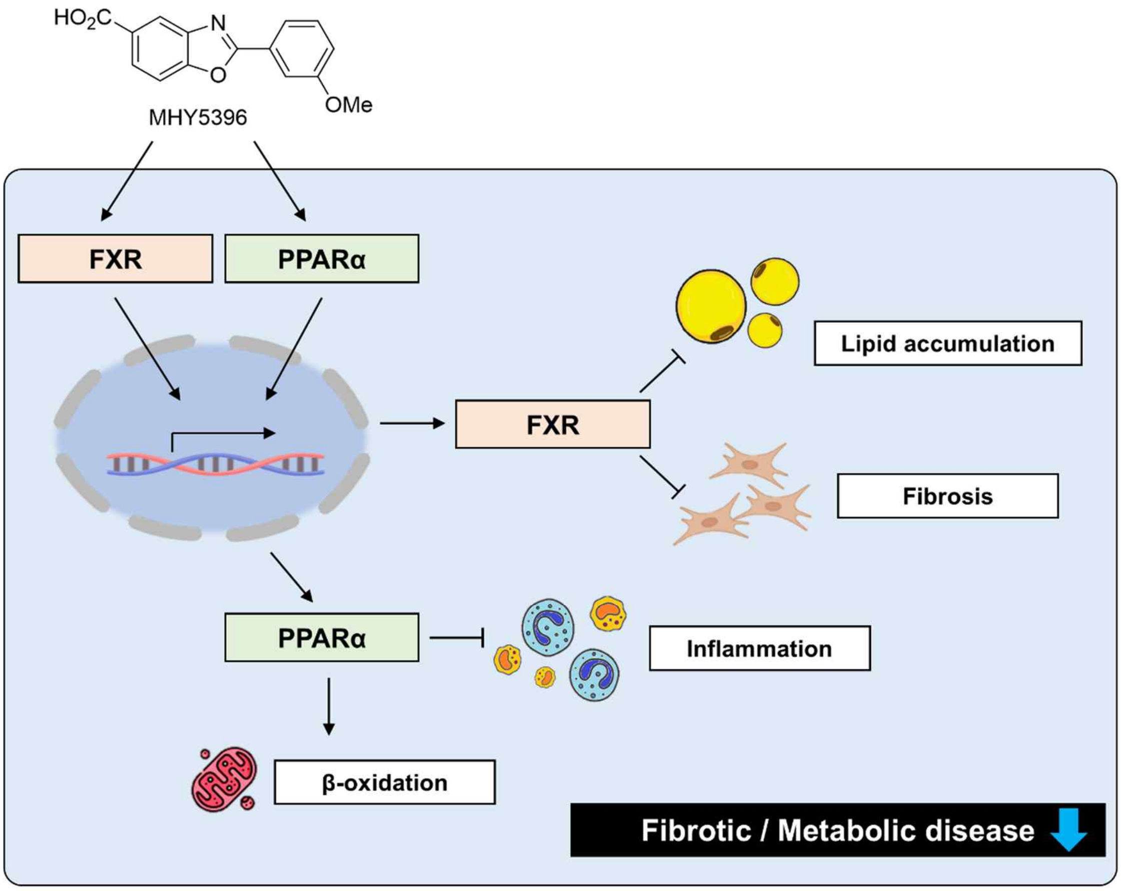

References cited in Supplementary information

1 Han DG, Seo SW, Choi E, Kim MS, Yoo JW, Jung Y, et al. Impact of route-dependent phase-II gut metabolism and enterohepatic circulation on the bioavailability and systemic disposition of resveratrol in rats and humans: A comprehensive whole body physiologically-based pharmacokinetic modeling. *Biomed Pharmacother* 2022;**151**:113141.

2 Hakooz N, Ito K, Rawden H, Gill H, Lemmers L, Boobis AR, et al. Determination of a human hepatic microsomal scaling factor for predicting in vivo drug clearance. *Pharm Res* 2006;**23**:533-9.

3 Yoon IS, Choi MK, Kim JS, Shim CK, Chung SJ, Kim DD. Pharmacokinetics and first-pass elimination of metoprolol in rats: contribution of intestinal first-pass extraction to low bioavailability of metoprolol. *Xenobiotica* 2011;**41**:243-51.

4 Sansen S, Yano JK, Reynald RL, Schoch GA, Griffin KJ, Stout CD, et al. Adaptations for the oxidation of polycyclic aromatic hydrocarbons exhibited by the structure of human P450 1A2. *J Biol Chem* 2007;**282**:14348-55.

5 Morris GM, Huey R, Lindstrom W, Sanner MF, Belew RK, Goodsell DS, et al. AutoDock4 and AutoDockTools4: Automated docking with selective receptor flexibility. *J Comput Chem* 2009;**30**:2785-91.

6 Sanner MF. Python: a programming language for software integration and development. *J Mol Graph Model* 1999;**17**:57-61.

7 Seo SW, Han DG, Baek YM, Park MC, Yoo JW, Jung Y, et al. Investigation of the factors responsible for the low oral bioavailability of alizarin using a sensitive LC-MS/MS method: In vitro, in situ, and in vivo evaluations. *Drug Dev Res* 2023;**84**:579-91.
